# Combinatorial immunotherapy with anti-ROR1 CAR NK cells and an IL-21 secreting oncolytic virus against neuroblastoma

**DOI:** 10.1016/j.omton.2024.200927

**Published:** 2024-12-21

**Authors:** Yaya Chu, Meijuan Tian, Uksha Saini, Jessica Ayala-Cuesta, Kayleigh Klose, Alyssa S. Mendelowitz, Keira Foley, Mehmet F. Ozkaynak, Wen Luo, Timothy P. Cripe, Dean A. Lee, Kevin A. Cassady, Mitchell S. Cairo

**Affiliations:** 1 Department of Pediatrics, New York Medical College, Valhalla, NY 10595, USA; 2 Center for Childhood Cancer and Blood Diseases, Nationwide Children’s Hospital, The Ohio State University, Columbus, OH 43210, USA; 3 Department of Cell Biology & Anatomy, New York Medical College, Valhalla, NY 10595, USA; 4 Department of Pathology, Microbiology & Immunology, New York Medical College, Valhalla, NY, USA; 5 Department of Medicine, New York Medical College, Valhalla, NY 10595, USA

**Keywords:** neuroblastoma, chimeric antigen receptor, CAR, ROR1, natural killer cells, oncolytic herpes simplex virus, interleukin-21, IL-21

## Abstract

Children with recurrent/metastatic neuroblastoma (NB) have a dismal survival (<25%). Novel therapies are desperately needed. Receptor tyrosine kinase-like orphan receptor 1 (ROR1) is highly expressed on NB. C021 is a selective oncolytic herpes simplex virus modified to overexpress human interleukin-21 (hIL-21), a cytokine that enhances natural killer (NK) cell cytotoxicity. In the current study, we successfully engineered *ex*-*vivo*-expanded NK cells to express a chimeric antigen receptor (CAR) against ROR1 using mRNA electroporation and investigated the efficacy of anti-ROR1-CAR-NK cells combined with C021 in targeting ROR1^+^ NB. We found that C021-infected NB cells secreted hIL-21 *in vitro* and *in vivo*. Compared to the non-cytokine-secreting parental virus C134, C021 significantly enhanced the *in vitro* cytotoxicity (*p* < 0.05) of anti-ROR1-CAR-NK cells with increased interferon (IFN)-γ (*p* < 0.05), granzyme B (*p* < 0.05), and perforin (*p* < 0.05) secretion against NB cells. Furthermore, the combination of C021 and anti-ROR1-CAR-NK cells significantly extended the survival of human NB xenografted NSG mice compared to controls (mock NK, ROR1-CAR-NK, C134, C021, C134+ROR1-CAR-NK, and C021+mock NK). Our results suggest that cytokine-secreting oncolytic virus in combination with CAR-NK cells is a novel, effective immunotherapeutic approach for high-risk NB.

## Graphical abstract

**Figure:**
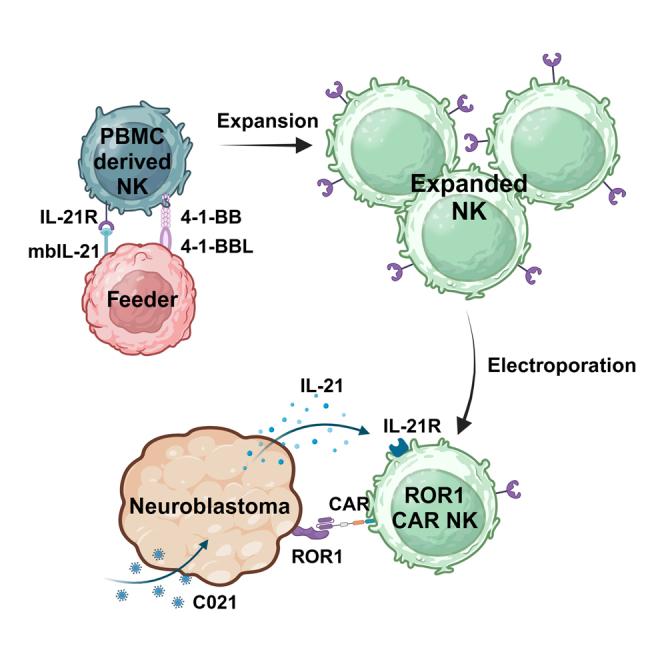

## Teaser

Prognosis remains poor for patients with high-risk neuroblastoma (NB), largely secondary to NB TME chemoradiotherapy resistance. Cairo and colleagues developed a CAR NK cell targeting ROR1 and demonstrated the anti-tumor efficacy of the combination of an oncolytic virus engineered to secrete IL-21 (C021) with anti-ROR1-CAR-NK cells against NB.

## Introduction

Neuroblastoma (NB) stands as the most prevalent and fatal extracranial solid tumor affecting children, contributing to approximately 15% of childhood cancer fatalities.^1^ Despite sustained efforts over several decades utilizing multi-chemotherapy, surgical resection, radiotherapy, autologous stem cell transplant, and targeted immunotherapy, survival for patients with high-risk NB remains dismal.^2^ There is an urgent and unmet need for the development of novel treatments for these patients. Receptor tyrosine kinase-like orphan receptor 1 (ROR1) belongs to the receptor tyrosine kinase family^3^ and has gained attention for its expression in several types of cancer, including NB, and its potential role as a therapeutic target.^4^

Natural killer (NK) cells provide the first line of defense against tumor cells.^5^ Clinical trials (ClinicalTrials.gov: NCT02573896 and NCT04211675) are underway to assess the safety and efficacy of NK cell therapy alone or in combination with other modalities in treating patients with high-risk NB. However, NK therapy has current limitations, including small numbers of cells, poor cellular function, and/or decreased persistence *in vivo.*^6^ Cytokines, however, have the potential to overcome these limitations by inducing proliferation and expansion, enhancing functional activity, and promoting persistence.

Interleukin-21 (IL-21) has the capability to promote the proliferation and function of NK cells.^7^ In preclinical studies, IL-21 has demonstrated potent anti-tumor effects through mechanisms including the induction of interferon (IFN)-γ and the activation of NK and cytotoxic T cells.^8^^,^^9^ Clinical trials have indicated promise for IL-21 as an immunotherapeutic agent in the treatment of patients with metastatic melanoma, showcasing both a favorable safety profile and notable anti-tumor activity.^10^^,^^11^

The oncolytic virus is a subtype of lytic virus designed to selectively infect and eliminate cancer but not normal cells.^12^ Among the oncolytic viruses, oncolytic herpes simplex viruses (oHSVs) stand out, having advanced to phase 3 clinical trials.^12^^,^^13^ oHSV possesses several therapeutic advantages, including its ability to infect various types of cancer cells and its ease of genetic modification.^14^ The next-generation oHSV C134 has been genetically engineered to express a tumor-associated antigen (TAA), EphA2, to enhance TAA immune recognition and improve the anti-tumor activity of oHSV.^15^

In this study, we leveraged the C134-based virus and modified it to incorporate the expression of human IL-21 (hIL-21; C021). We hypothesized that intratumoral delivery of IL-21 by C021 would amplify the local anti-tumor efficacy of anti-ROR1-CAR-NK cells against NB.

## Results

**Figure 1:**
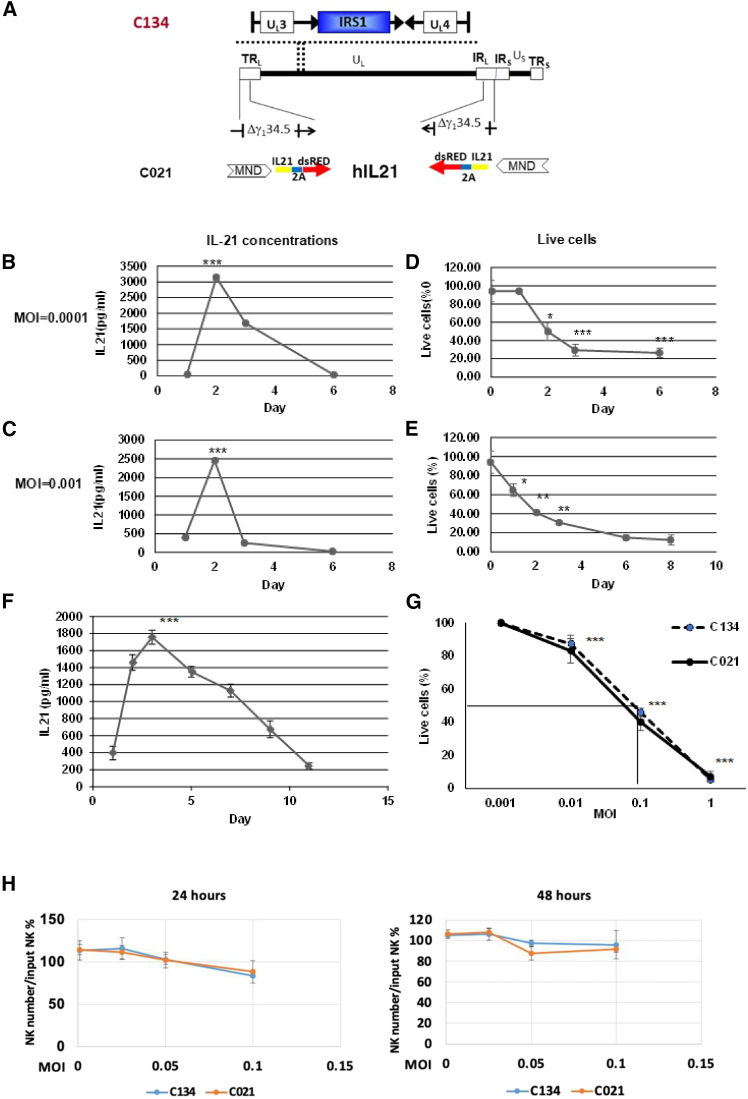
The effects of C134 and C021 on NB cells and *ex*-*vivo*-expanded NK cells *in vitro* (A) Schematic representation of C134 and C021 constructs. C021 contains the hIL-21 gene and dsRed (linked by a 2A element) within both copies of the γ_1_34.5 deletion locus of C134. (B) C021-infected CHLA-255 cells secreted significantly higher levels of hIL-21 on day 2 at MOI = 0.0001 (*p* < 0.001) compared to day 1, 3, or 6. *n* = 3. In this and the subsequent panels, markers represent the mean values, error bars indicate the standard deviation (SD) of triplicate samples in a representative experiment. The same trend was seen in three independent biological replicates. Results were compared using the two-tailed Student t-test with *p* < 0.05 considered as significant. (C) C021-infected CHLA-255 cells secreted significantly higher levels of hIL-21 on day 2 at MOI = 0.001 (*p* < 0.001) compared to day 1, 3, or 6. *n* = 3. (D) C021 significantly reduced the viability of CHLA-255 cells at day 2, 3, or 6 (*p* < 0.001) compared to day 1 at MOI = 0.0001. *n* = 3. (E) C021 significantly reduced the viability of CHLA-255 cells at day 2, 3, or 6 (*p* < 0.001) compared to day 1 at MOI = 0.001. *n* = 3. (F) At MOI = 0.025, C021-infected SKNFI cells secreted significantly higher levels of hIL-21 on day 3 (*p* < 0.001) compared to day 1, 2, 5, 7, 9, or 11. *n* = 3. (G) Both C134 and C021 infections significantly reduced the viability of SKNFI cells at 24 h at MOI = 0.01, 0.1, or 1 compared to MOI = 0.001 (*p* < 0.001). *n* = 3. (H) *Ex*-*vivo*-expanded NK cells were incubated with C134 or C021 at the indicated MOI. NK viability was measured at 24 and 48 h by CellTiter 96 AQueous one solution cell proliferation assay. *n* = 3. ∗*p* < 0.05 and ∗∗∗*p* < 0.001.

We modified C134 oncolytic virus to express hIL-21 (C021) (Figure 1A) and infected CHLA-255 NB cells with C021 utilizing a range of multiplicities of infection (MOIs) (Figures S1A–S1C). We found that infected CHLA-255 cells secreted significantly higher levels of hIL-21 at day 2 post-infection at MOI = 0.0001 (Figure 1B) (*p* < 0.001) or 0.001 (Figure 1C) (*p* < 0.001) compared to day 1, 3, or 6. In addition, C021 infection, at MOI = 0.0001 or 0.001, significantly reduced the viability of CHLA-255 cells (*p* < 0.001) (Figures 1D and 1E). Similarly, at MOI = 0.025, C021-infected SKNFI NB cells secreted higher levels of hIL-21 at day 3 post-infection (*p* < 0.001 compared to day 1, 2, 5, 7, 9, or 11) (Figure 1F), and C021 infection at MOI = 0.01, 0.1 or 1 significantly reduced the viability of SKNFI cells (*p* < 0.001 compared to MOI = 0.001) (Figure 1G). Notably, we observed no significant difference between C134 and C021 infections on the viability of SKNFI cells (Figure 1G). Neither C134 nor C021 reduced the viability of NK cells at the tested MOI (Figure 1H).

**Figure 2:**
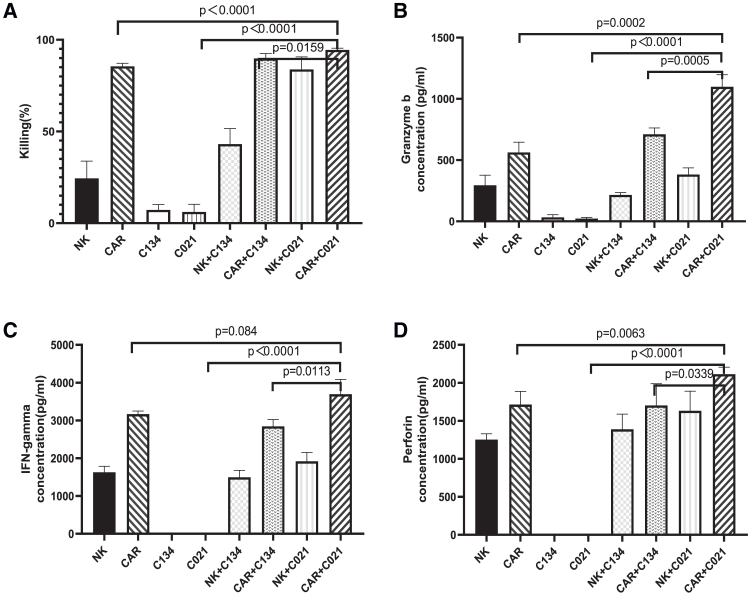
C021 significantly enhanced *in vitro* cytotoxicity with the enhanced release of granzyme B, IFN-γ, and perforin of anti-ROR1-CAR-NK cells against NB cells (A) Mock NK or anti-ROR1-CAR-NK cells (CAR) were incubated with SKNFI cells at E:T = 3:1 with or without C134 or C021 (MOI = 0.025) for 24 h. The percentage of killing of SKNFI cells was measured by Britelite plus reporter gene assay. C021 significantly enhanced the *in vitro* cytotoxicity of anti-ROR1-CAR-NK cells against SKNFI cells compared to controls. In this and the subsequent panels, columns represent the mean values, error bars indicate the standard deviation (SD) of triplicate samples in a representative experiment. The same trend was seen in three independent biological replicates. Results were compared using the two-tailed Student t-test with *p* < 0.05 considered as significant. (B–D) After 24 h co-culture under the condition as described in (A), the supernatants were collected for ELISAs to determine the released granzyme B (B), IFN-γ (C), and perforin (D) levels. (E) Mock NK or anti-ROR1-CAR-NK cells (CAR) were incubated with CHLA-255 cells at E:T = 1:1 with or without C134 or C021 (MOI = 0.001) for 24 h. The percentage of killing of CHLA-255 cells was measured by Britelite plus reporter gene assay. C021 significantly enhanced the *in vitro* cytotoxicity of anti-ROR1-CAR-NK cells against CHLA-255 cells compared to controls (*p* = 0.0286 vs. CAR and CAR+C134, *p* < 0.0001 vs. C021). (F–H) After 24 h co-culture under the condition as described in (E), the supernatants were collected for ELISAs to determine the released granzyme B (F), IFN-γ (G), and perforin (H) levels. *n* = 4.

We next electroporated expanded NK cells^16^ with anti-ROR1-CAR mRNA to generate anti-ROR1-CAR-NK cells (Figure S2) and investigated if C021 would enhance the *in vitro* cytotoxicity of anti-ROR1-CAR-NK cells against NB cells. Mock NK or anti-ROR1-CAR-NK cells were incubated with SKNFI cells at effector -to-target ratio (E:T) = 3:1 with or without C134 or C021 (MOI = 0.025) for 24 h. C021 again induced IL-21 secretion (Figure S3) and significantly enhanced the *in vitro* cytotoxicity of anti-ROR1-CAR-NK cells against SKNFI cells compared to all controls (CAR-NK [*p* < 0.0001], C021 [*p* < 0.0001], CAR-NK+C134 [*p* = 0.0159]) (Figure 2A). Consistent with the enhanced *in vitro* cytotoxicity, we found that C021 significantly enhanced the secretion of granzyme B (Figure 2B) (*p* = 0.0002 vs. CAR, *p* < 0.0001 vs. C021, *p* = 0.0005 vs. CAR+C134), IFN-γ (Figure 2C) (*p* = 0.084 vs. CAR, *p* < 0.0001 vs. C021, *p* = 0.0113 vs. CAR+C134), and perforin (Figure 2D) (*p* = 0.0063 vs. CAR, *p* < 0.0001 vs. C021, *p* = 0.0339 vs. CAR+C134) compared to all controls. Similarly, C021 (MOI = 0.001) significantly enhanced the *in vitro* cytotoxicity of anti-ROR1-CAR-NK cells against CHLA-255 cells at an E:T ratio of 1:1 (Figure 2E) (*p* < 0.0001, *p* = 0.0286) with significantly enhanced secretion of granzyme B (Figure 2F) (*p* < 0.0001), IFN-γ (Figure 2G) (*p* < 0.0001, *p* = 0.0001, *p* = 0.0385), and perforin (Figure 2H) (*p* < 0.0001, *p* = 0.0286, *p* = 0.0403) compared to all controls.

**Figure 3:**
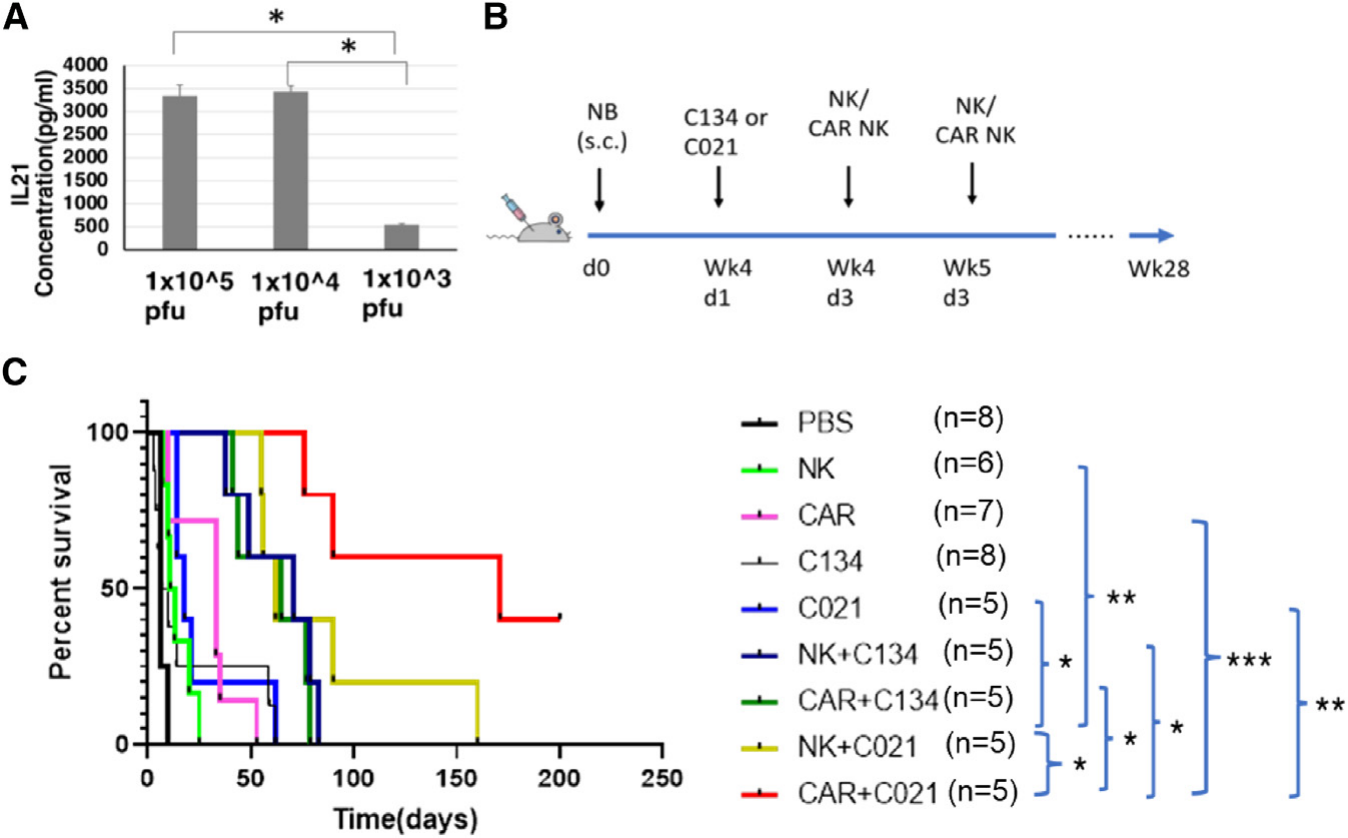
The combination of anti-ROR1 CAR NK+C021 significantly extended the survival of NB xenografted NSG mice (A) hIL-21 expression in C021-injected NB xenograft tumors. 2 × 10^7^ of CHLA-255-Luc cells were subcutaneously injected into the right flank of NSG mice. After the tumor diameter reached 1 ± 0.3 cm, one dose of 1 × 10^3^, 10^4^, or 10^5^ PFU C021 was intratumorally injected into CHLA-255-Luc xenografted NSG mice. Two days later, tumors were collected and homogenized in 1 mL RPMI1640 medium. The tumor samples were centrifuged, and the supernatant was collected and used for IL-21 ELISAs. Columns represent the mean values, error bars indicate the standard deviation (SD) of all the samples. Results were compared using the two-tailed Student t-test with *p* < 0.05 considered as significant. (B) Experimental schema. 2 × 10^7^ of CHLA-255-Luc cells were subcutaneously injected into the right flank of NSG mice. After the tumor diameter reached 1 ± 0.3 cm, one dose of PBS or 1 × 10^4^ PFU C134 or C021 was intratumorally injected into CHLA-255-Luc xenografted mice. PBS, 5 × 10^6^ expanded peripheral blood natural killer (exPBNK) cells, or 5 × 10^6^ anti-ROR1-CAR-NK cells were intraperitoneally injected to each mouse 2 or 9 days after viral injection. (C) The Kaplan-Meier survival curves of mice receiving treatment are shown using animal sacrifice as the terminal event. Survival curves were analyzed using the log rank (Mantel-Cox) test. The mice treated with anti-ROR1-CAR-NK+C021 (*n* = 5) significantly extended the survival of CHLA255 xenografted mice as compared to the control groups, which were treated with mock NK+C021 (*n* = 5, *p* < 0.05), anti-ROR1-CAR-NK cells alone (*n* = 5, *p* < 0.05), or C021 alone (*n* = 5, *p* < 0.01). NK, mock NK; CAR, anti-ROR1-CAR-NK. ∗*p* < 0.05, ∗∗*p* < 0.01, and ∗∗∗*p* < 0.001.

Next, we investigated if C021-infected NB xenograft tumors would secrete hIL-21 *in vivo*. We injected CHLA-255 cells subcutaneously into NSG mice and injected C021 intratumorally once the tumor was established. We found that the tumors infected with 1 × 10^4^ or 1 × 10^5^ PFU/mouse C021 secreted significantly higher levels of hIL-21 (*p* < 0.05) compared to tumors infected with 1 × 10^3^ PFU/mouse (Figure 3A), and the high IL-21 level in the tumors (Figure S4) lasted longer than in cell culture (Figure 1C).

To investigate if the combination of anti-ROR1-CAR-NK cells and C021 has advantages over anti-ROR1-CAR-NK cells alone or C021 alone in limiting NB tumor growth and improving mice survival, we xenografted luciferase-expressing CHLA-255 cells subcutaneously into NSG mice and treated the animals with PBS or 1 × 10^4^ PFU C134 or C021 intratumorally (once) followed by intraperitoneal injection of PBS or 5 × 10^6^ mock NK or ROR1-CAR-NK cells (twice: 2 and 9 days after viral injection) (Figures 3B and S5). The Kaplan-Meier survival curves showed that the mice treated with anti-ROR1-CAR-NK cells+C021 (*n* = 5) had significantly extended survival as compared to the control groups, which were treated with mock NK+C021 (*n* = 5, *p* < 0.05), anti-ROR1-CAR-NK cells alone (*n* = 5, *p* < 0.05), or C021 alone (*n* = 5, *p* < 0.01) (Figure 3C).

## Discussion

Limited therapeutic options for high-risk NB highlight the pressing necessity for the development and implementation of innovative strategies to improve outcomes in this vulnerable patient population. In a parallel study, we demonstrated that ROR1 is highly expressed on NB and developed anti-ROR1-CAR-modified NK cells, and these anti-ROR1- CAR-NK cells showed significantly enhanced *in vitro* and *in vivo* anti-tumor effect against NB.^17^ In the current study, our data demonstrated the anti-NB efficacy of the combination of an oncolytic virus engineered to secrete hIL-21 (C021) with anti-ROR1-CAR-NK cells.

Oncolytic viral therapy represents a promising avenue in the treatment of high-risk NB.^18^ NB cell lines and human primary NB specimens expressed high levels of CD111, the primary entry protein of oHSV.^19^ To reduce neurotoxicity and ensure replication in actively dividing cancer cells, but not normal (growth-arrested) cells, the *γ*_1_34.5 gene, which encodes the protein 34.5 (ICP34.5), was deleted in C134 and C021.^13^ This is consistent with our finding that both C134 and C021 efficiently lysed NB cells but not NK cells (Figure 1).

IL-21 shares the common γ chain as IL-2 and IL-15 and plays a crucial role in promoting NK proliferation and maturation from bone marrow.^20^^,^^21^ The Lee laboratory developed a genetically engineered antigen-presenting cell (K562) expressing membrane-bound IL-21 and 4-1BBL (K562-mbIL21-41BBL) that expands NK cells out of peripheral blood mononuclear cells (PBMCs).^16^ The donor-derived haploidentical NK cells expanded utilizing the K562-mbIL21-41BBL cells were safe, with significantly improved NK cell number and function, and improved survival in patients with myeloid malignancies.^22^^,^^23^ Additionally, IL-21 helps sustain NK cell survival and memory-like responses, making it a key cytokine in modulating the overall effectiveness of NK cell-mediated immune responses.^24^ IL-21 is critical to reverse the functions of exhausted NK cells.^25^ To our knowledge, this is the first preclinical study investigating the therapeutic potential of the combination of anti-ROR1-CAR-NK cells with IL-21 intratumorally delivered by oncolytic viruses in treating high-risk NB.

Systemic delivery of IL-21 to NB may be limited by the inability to reach adequate concentrations of IL-21 across the entire tumor. Local delivery of IL-21 through oncolytic viruses offers several advantages, such as targeted delivery to tumor cells, reduced systemic toxicity, increased immune cell infiltration in tumor mass, enhanced therapeutic efficacy, a modified tumor microenvironment (TME), and immune-cell efficacy promotion.^12^ Our data showed that C021 induced a high level of IL-21 secretion in the NB tumors, which sustained for around 6 days (Figure S4), demonstrating the successful local delivery of IL-21. Since C021 is modified to replicate specifically in tumor cells, this strategy facilitates efficient oncolysis of NB cells (Figure 1G) while minimizing probability of adverse off-target effects. Systemically delivered oncolytic viruses can be neutralized by the pre-existing or therapy-induced neutralizing antibodies; however, this risk can be minimized with local delivery. Although our *in vivo* study has a limitation in that it was investigated in a small set of animals, our finding that the combinatorial therapy of NK/anti-ROR1-CAR-NK cells with C021 significantly improved the survival of NB xenografted mice indicates that C021 shaped the TME and promoted NK/CAR-NK therapeutic efficacy. Further investigations with larger sample sizes will be necessary to prove this hypothesis.

In conclusion, our results demonstrate the significant anti-tumor efficacy of the combination of oHSV C021 with anti-ROR1-CAR-NK cells targeting NB cells *in vitro* and *in vivo*. Further research and clinical trials are essential to validate the safety and efficacy of this combination in treating high-risk NB.

## Materials and methods

### Anti-ROR1-CAR-NK generation

The anti-ROR1-CAR mRNA was synthesized *in vitro* using the mMESSAGE mMACHINE T7 Ultra kit as we previously described.^26^ Expanded NK cells were electroporated with anti-ROR1-CAR mRNA (1 μg mRNA per 1 × 10^6^ NK cells in EPB5 buffer) using the NK3 protocol of the MaxCyte GT electroporation System (Maxcyte, Rockville, MD, USA).

Additional methods are detailed in the supplemental information.

## Data and code availability

Data are available upon reasonable request.

## Acknowledgments

The authors would like to thank Erin Morris, BSN, and Virginia Davenport, RN, for their excellent assistance with the preparation of this manuscript and Janet Ayello, MS, for her assistance with purchasing research reagents. The research for this study was primary supported by a grant from the 10.13039/100000002NIH (1U54 CA232561-01A1; M.S.C., D.A.L., and T.P.C.) and additional support from the 10.13039/100000902Pediatric Cancer Research Foundation (M.S.C.) and the Children Cancer Foundation (M.S.C.).

## Author contributions

Conceptualization, Y.C. and M.S.C.; visualization, Y.C. and M.T.; writing – original draft, Y.C., M.T., and M.S.C.; writing – review & editing, Y.C., M.T., U.S., J.A.-C., K.K., A.S.M., K.F., M.F.O., W.L., T.P.C., D.A.L., K.A.C., and M.S.C.; methodology, Y.C., M.T., U.S., and K.A.C.; formal analysis, Y.C., M.T., U.S., and K.A.C.; data curation, Y.C. and M.T.; validation, Y.C.; administrative, technical, and material support, T.P.C., D.A.L., K.A.C., and M.S.C.; funding, T.P.C., K.A.C., and M.S.C.

## Declaration of interests

This work was presented in part at the Pediatric Transplantation & Cellular Therapy Consortium (2023), Fort Worth, TX; International Society for Cell & Gene Therapy (2022), San Francisco, CA; and Transplantation & Cellular Therapy Meetings of the American Society for Transplantation and Cellular Therapy (2022), Salt Lake City, Utah. M.S.C. has served as a consultant for Jazz Pharmaceuticals, Omeros Pharmaceuticals, Servier Pharmaceuticals, Abbvie, and Novartis Pharmaceuticals; on speakers bureau for Jazz Pharmaceuticals, Servier Pharmaceuticals, Amgen, Inc., Sanofi, and Sobi; and on the advisory board for Astra Zeneca and has received research funding from Celularity, Merck, Miltenyi Biotec, Servier, Omeros, Jazz, and Janssen. D.A.L. reports personal fees and other fees from Kiadis Pharma, CytoSen Therapeutics, Courier Therapeutics, and Caribou Biosciences outside the submitted work. In addition, D.A.L. has a patent broadly related to NK cell therapy of cancer with royalties paid to Kiadis Pharma. T.P.C. recently served as a one-time consultant to Blueprint, Incyte, and Oncopeptides and a DSMB chair for SpringWorks and is a cofounder of Vironexis Biotherapeutics, Inc.

## Supplemental information

Document S1. Figures S1–S5 and supplemental materials and methods

Document S2. Article plus supplemental information
