# Supplementary material for: Combinatorial immunotherapy with anti-ROR1 CAR NK cells and an IL-21 secreting oncolytic virus against neuroblastoma
Source: Mol Ther Oncol. 2024 Dec 21;33(1):200927. doi: 10.1016/j.omton.2024.200927 (PMC11783442; doi:10.1016/j.omton.2024.200927)
Supplement: Document S1. Figures S1–S5 and supplemental materials and methods [file mmc1.pdf]

OMTON, Volume *33*

Supplemental information

Combinatorial immunotherapy with anti-ROR1 CAR NK cells and an IL-21 secreting oncolytic virus against neuroblastoma

Yaya Chu, Meijuan Tian, Uksha Saini, Jessica Ayala-Cuesta, Kayleigh Klose, Alyssa S. Mendelowitz, Keira Foley, Mehmet F. Ozkaynak, Wen Luo, Timothy P. Cripe, Dean A. Lee, Kevin A. Cassady, and Mitchell S. Cairo

Supplemental Methods

Cell lines, viruses, and reagents

SKNFI cells were purchased from the American Type Culture Collection, Gaithersburg, MD, USA. The luciferase expressing CHLA-255 cells were generously provided by Robert Seeger, MD from Children's Hospital Los Angeles, CA, USA. Anti-ROR1 scFv was generously provided by Stanley Riddell, MD from Fred Hutchinson Cancer Center, Seattle, WA, USA. Leukocytes were obtained after informed consent from healthy donors at the New York Blood Center, New York, New York, USA. Peripheral blood mononuclear cells (PBMNC) were obtained by Ficoll gradient (Cytiva, Marlborough, MA, USA) separation as we previously described.[1] Recombinants C134 has been described previously.[2,3] Briefly, C134 is a $\Delta\gamma_1 34.5$ virus that contains the HCMV IRS1 gene under control of the CMV IE promoter in the U_L3/ U_L4 intergenic region and has been described previously.[2] C154 is an EGFP-expressing version of C134 with EGFP encoded in the $\gamma_1 34.5$ locus flanked by engineered Pac I restriction sites. C021 was created using the PacI homologous recombination method previously described (Figure 1A).[4] In brief the Pac I digested C154 viral DNA was co-transfected with linearized targeting plasmid pCK1238, encoding the Egr1 promoter driven hIL21 coding F2A and dsRED coding domain. C021 was then purified by loss of GFP and gain of dsRED expression by serial plaque selection. Viruses were confirmed genetically by DNA hybridization studies and IL21 expression was confirmed by ELISA.

NK cell expansion

PBMNCs were stimulated with irradiated genetically modified K562-mbIL21- 41BBL cells as we previously described.[5] Expanded NK cells were isolated by negative selection using Miltenyi

NK cell isolation kit (Miltenyi Biotec, Cambridge, MA, USA) as we have previously described.[1] Expanded purified NK cells were cultured in Gibco RPMI 1640 medium (Thermofisher) supplemented with 10% heat-inactivated FBS (Thermofisher), 100 U/mL penicillin, 100 μg/mL streptomycin (Thermofisher), 4 mmol/L glutamine (Thermofisher), and 50 IU/ml IL-2.

Anti-ROR1-CAR-NK generation

The ROR1 CAR was generously provided by Stanley Riddell MD (Fred Hutchinson Cancer Research Center) and was previously reported.[6,7] The CAR possessed a murine CD8α signal peptide (UniProt: P01731, aa1–27), R11 scFv, modified human IgG4 long spacer with 4/2NQ mutations (Hudecek et al., 2015), murine CD28 transmembrane (UniProt: P31041, aa151–177), murine 4–1BB (UniProt: P20334, aa211–256), murine CD3ζ (UniProt: P24161, aa52–164). The anti-ROR1-CAR mRNA was synthesized *in-vitro* using the mMESSAGE mMACHINE T7 Ultra kit as we previously described.[1] Expanded NK cells were electroporated with anti-ROR1-CAR mRNA using the MaxCyte GT® electroporation System (Maxcyte Inc., Rockville, MD, USA). Anti-ROR1-CAR mRNA electroporation efficacy was evaluated by flow cytometry analysis using an FITC-conjugated goat anti-mouse IgG, $F(ab')_2$ fragment-specific antibody. Expanded NK cells electroporated with H2O (mock NK) were used as a control.

Luciferase based *in-vitro* cytotoxicity

Expanded NK or anti-ROR1-CAR-NK cells were incubated with SKNF1-Luc cells at the effector:target (E:T) ratio=3:1 with or without C134 or C021 (MOI = 0.025) at 37°C for overnight. NK or anti-ROR1-CAR-NK cells were incubated with CHLA -255-Luc cells E:T ratio=1:1 with or without C134 or C021 (MOI = 0.001) in RPMI media at 37°C for overnight.

Cytotoxicity was determined by Britelite plus reporter gene assay (PerkinElmer, 6066761) without cell lysis. After adding luciferin, the luminescence emission of viable cells was measured by a plate reader (Molecular devices, Filter max F5 microplate reader) within 15 minutes. Cytotoxicity was calculated as follows: % cytotoxicity= (Luminescence release (untreated tumor cells)- Luminescence release (treated tumor cells)) / Luminescence release (untreated tumor cells)) ×100.

MTS assays

PBMNCs were stimulated with irradiated genetically modified K562-mbIL21 - 41BBL cells for 2-3 weeks. Purified expanded NK cells were cultured in medium with C134 or C021 at different MOI for 24 hours or 48 hours. CellTiter 96 AQueous one solution cell proliferation assay (Promega, Madison, WI, USA) was used to determine the number of proliferating viable cells following the manufacturer instructions as we previously described.[8] Spectrophotometrical absorbance was measured using a multifilter plate reader (Molecular Device, San Jose, CA, USA) at OD490.

Enzyme-linked immunosorbent assay (ELISA)

Human granzyme B (Mabtech Cat#3485-1H-20), perforin (Mabtech Cat#3465-1H-20), or IFN-γ (Mabtech Cat#3420-1H-20) were measured by ELISA assays. Greiner Bio-One 96 well high binding standard ELISA microplates (Cat# 655061) were coated with 100 µl of human granzyme B, perforin, or IFN-γ (2 µg/ml ,4 µg/ml, 2µg/ml in phosphate-buffered saline (PBS), respectively) by incubating plates overnight at 4 °C. Wells were then washed twice with 200 µl wash buffer (0.05% Tween-20 in PBS) and blocked with 250 µl blocking buffer (0.1% BSA in

PBS) for 1 hour at room temperature. Samples or standards were added to the wells (100 µl per well) in technical duplicates or triplicates, and plates were incubated for 2 hours at room temperature and washed three times. Biotin conjugated antibodies (1µg/ml, 100 µl) were added and incubated for 1 hour at room temperature. Following 3 times of wash, 100 µl of horseradish peroxidase- diluted 1:1,000 was added to the wells and plates were incubated for 1 hour at room temperature. The wells were washed three times and incubated with 100 µl of TMB substrate solution (Invitrogen, Waltham, MS, USA) for 2-5 mins. The reaction was stopped by adding 100 µl of 2 N sulfuric acid, and plates were read at 450 nm using the FilterMax F5 (Molecular devices) plate reader.

Xenograft models

Six- to eight-week-old NSG mice were purchased from the Jackson Laboratory (Bar Harbor, ME, USA). Mice were bred, treated, and maintained under pathogen-free conditions in-house under New York Medical College Institutional Animal Care and Use Committee-approved protocols. CHLA-255-Luc cells ($2x10^7$) were subcutaneously injected to the right flanks of NSG mice. After the tumor diameter reached 1±0.3 cm, the mice were separated into 9 groups: 1) Mock NK, 2) anti-ROR1-CAR-NK, 3) C134, 4) C021, 5) Mock NK+ C134, 6) Mock NK+C021, 7) anti-ROR1-CAR-NK+C134, 8) anti-ROR1-CAR-NK+C021, and 9) PBS. $1x10^4$ pfu C134 or C021 or PBS was intratumorally injected on day 1 of the treatment (7 days after tumor cell injection). Two days and nine days later, $5x10^6$ NK or anti-ROR1-CAR-NK cells or PBS were intraperitoneally injected. Tumor engraftment and progression were evaluated using the Xenogen IVIS-200 system (PerkinElmer, Shelton, CT, USA) as we have previously described.[1] Tumor

size was measured by a caliper. Mice were followed until death or sacrificed if the diameter of tumor reached 2 cm.

Statistical analyses

Statistical analyses were performed using the Prism program 10.0 (GraphPad Software, Inc.). Average values are reported as the mean ± SD. Results were compared using the two-tailed Student t-test with $p < 0.05$ considered as significant. Probability of survival in animal studies was determined by the Kaplan-Meier method using the Prism program 10.0 (GraphPad Software, Inc., La Jolla, CA, USA). Survival rates were compared using the log-rank Mantel–Cox test.

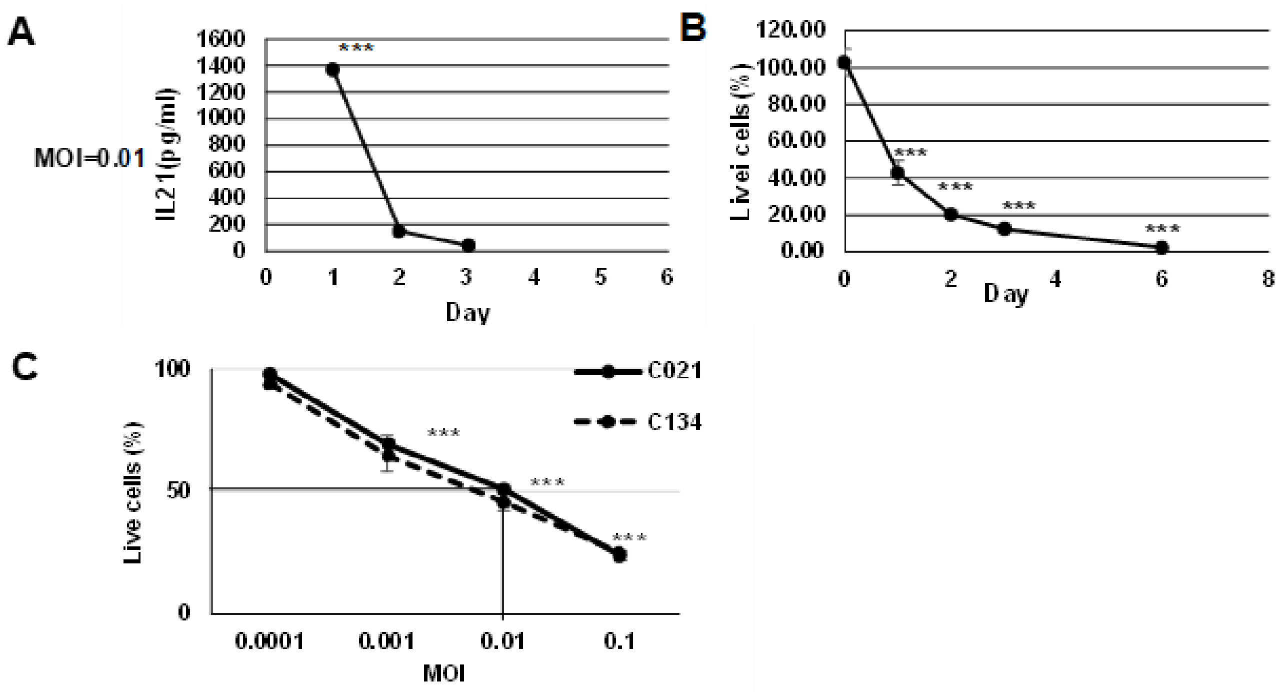

Figure S1. The effects of C134 and C021 on NB cells and ex-vivo expanded NK cells in-vitro. (A) C021 infected CHLA-255 cells secreted significantly higher level of hIL-21 at day 1 at MOI = 0.01 ($p < 0.001$) compared to day 2 or day 3. n = 3 (B) C021 significantly reduced the viability of CHLA-255 cells at day 2, 3 or 6 ($p < 0.001$) compared to day 1 at MOI = 0.01. n = 3. (C) Both C134 and C021 infection significantly reduced the viability of CHLA-255 cells at 24 hours at MOI = 0.001, 0.01 or 0.1 compared to MOI = 0.0001 ($p < 0.001$). n = 3

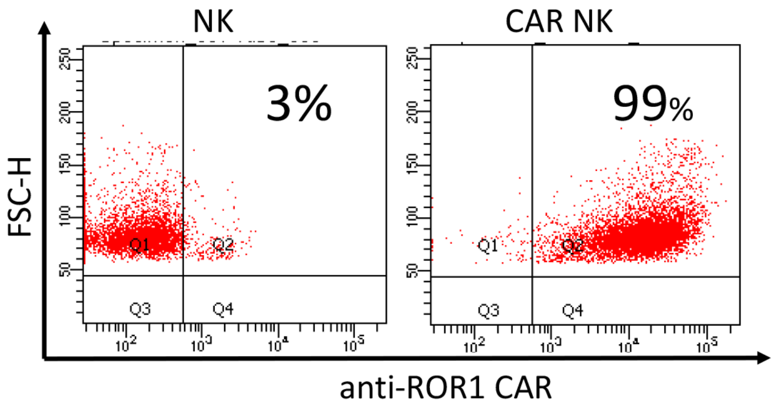

Figure S2. Representative flow cytometry dot plots show the anti-ROR1 CAR expression. The anti-ROR1 CAR mRNA or the anti-ROR1 CAR-CD19 mRNA was synthesized *in vitro* using the mMESSAGE mMACHINE T7 Ultra kit. Expanded NK cells were electroporated with anti-ROR1 CAR mRNA or the anti-ROR1 CAR-CD19 mRNA using the MaxCyte GT® electroporation System (Maxcyte Inc.). Anti-ROR1 CAR mRNA electroporation efficacy was evaluated by flow cytometry analysis at 24 hours post electroporation using an FITC-conjugated goat anti-mouse IgG, F(ab')$_2$ fragment-specific antibody. Expanded NK cells electroporated with H2O (mock NK) were used as control.

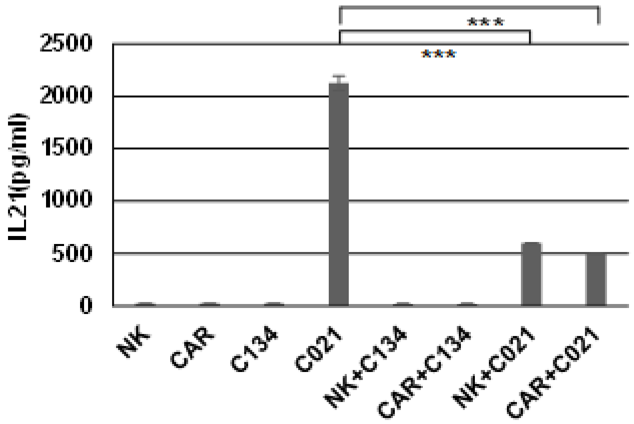

Figure S3. C021 release was influenced by cytotoxicity of NK or anti-ROR1-CAR-NK cells (CAR) cells. Mock NK or anti-ROR1-CAR-NK cells (CAR) were incubated with CHLA-255 cells at E:T=1:1 with or without the pretreatment of C134 or C021 (MOI = 0.001) for 24 hours. The supernatants were collected for ELISA assays to determine the hIL-21 release in each group.

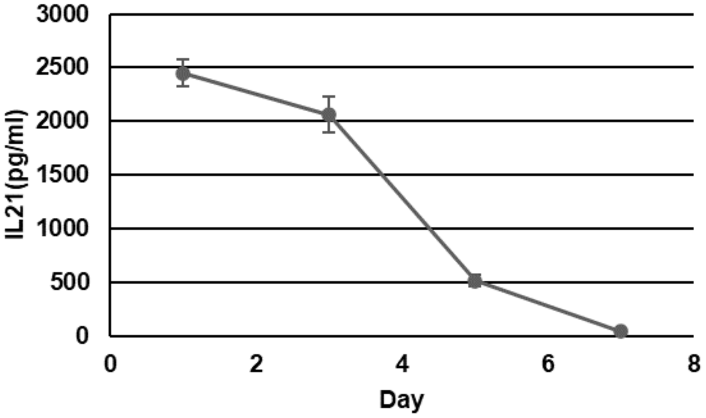

Figure S4. C021 infected NB cells in NB xenografts secreted hIL21. CHLA-255-Luc cells ($2x10^7$) were subcutaneously injected to the right flank in NSG mice. After the tumor diameter reached 1±0.3 cm, one dose of $1x10^4$ pfu C021 was intratumorally injected to the CHLA-255-Luc xenografted NSG mice. Tumors were collected and homogenized in 1ml RPMI1640 medium on day 1,3,5 and 7. The tumor samples were centrifuged, and the supernatant was collected and used for IL-21 ELISA assays. N=3.

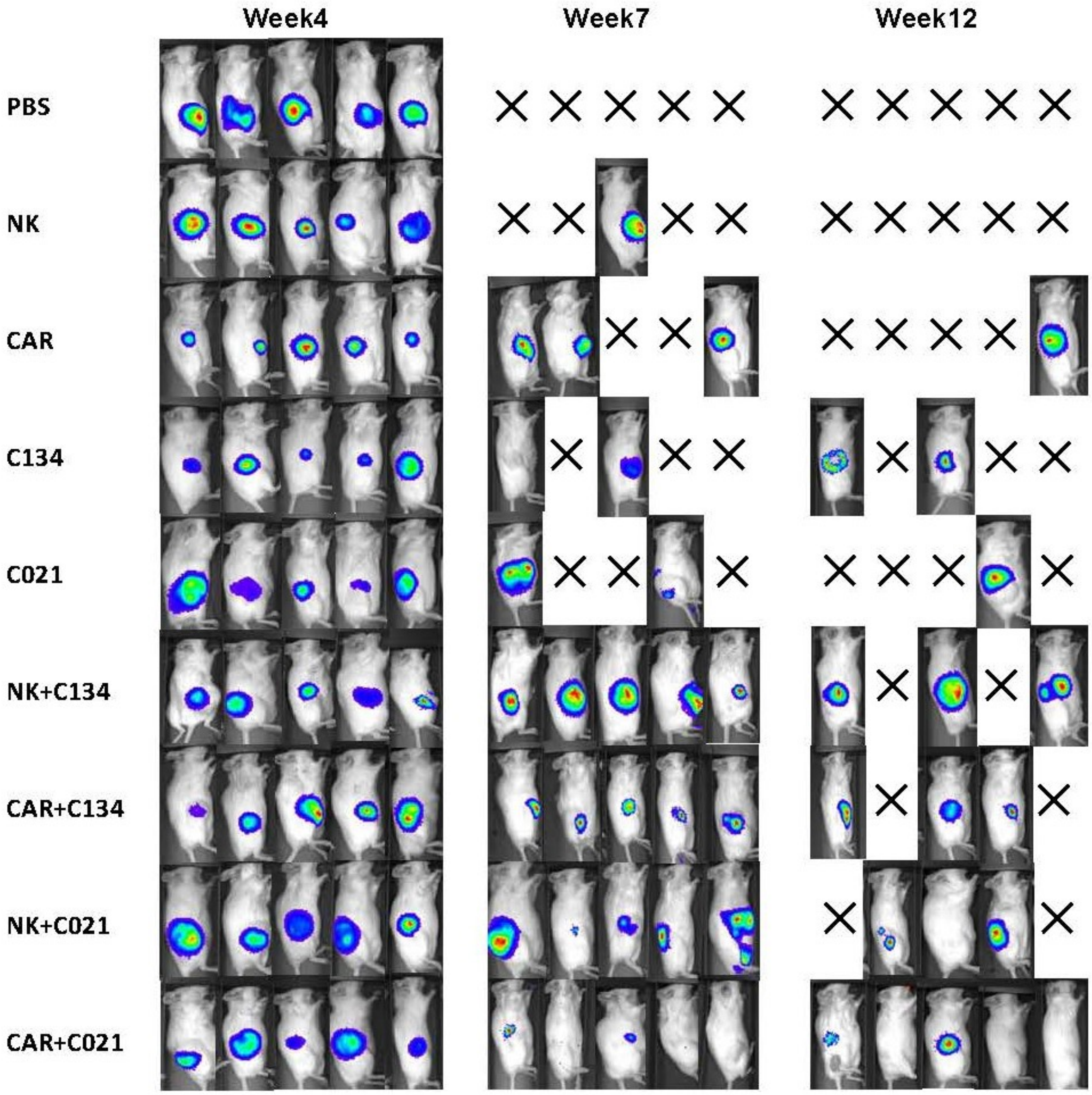

Figure S5. The combination of anti-ROR1 CAR NK + C021 significantly extended the survival of NB xenografted NSG mice. CHLA-255-Luc cells ($2x10^7$) were subcutaneously injected to the right flank in NSG mice. After the tumor diameter reached 1±0.3 cm, one dose of PBS, 1x104 pfu HSV virus or $5x10^6$ exPBNK /CAR cells were injected as mentioned in Fig.4. Representative bioluminescence images of mice of each group are shown at week4 (one day before treatment), week 7(2week post treatment) and week 12.

REFERENCES

1. Chu Y, Hochberg J, Yahr A, Ayello J, van de Ven C, Barth M, Czuczman M, Cairo MS. Targeting CD20+ Aggressive B-cell Non-Hodgkin Lymphoma by Anti-CD20 CAR mRNA-Modified Expanded Natural Killer Cells In Vitro and in NSG Mice. *Cancer Immunol Res*. 2015;3(4):333-344.
2. Cassady KA. Human cytomegalovirus TRS1 and IRS1 gene products block the double-stranded-RNA-activated host protein shutoff response induced by herpes simplex virus type 1 infection. *J Virol*. 2005;79(14):8707-8715.
3. Ghonime MG, Jackson J, Shah A, Roth J, Li M, Saunders U, Coleman J, Gillespie GY, Markert JM, Cassady KA. Chimeric HCMV/HSV-1 and Deltagamma(1)34.5 oncolytic herpes simplex virus elicit immune mediated antigliomal effect and antitumor memory. *Transl Oncol*. 2018;11(1):86-93.
4. Parker JN, Zheng X, Luckett W, Markert JM, Cassady KA. Strategies for the rapid construction of conditionally-replicating HSV-1 vectors expressing foreign genes as anticancer therapeutic agents. *Mol Pharm*. 2011;8(1):44-49.
5. Denman CJ, Senyukov VV, Somanchi SS, Phatarpekar PV, Kopp LM, Johnson JL, Singh H, Hurton L, Maiti SN, Huls MH, et al. Membrane-bound IL-21 promotes sustained ex vivo proliferation of human natural killer cells. *PLoS One*. 2012;7(1):e30264.
6. Srivastava S, Salter AI, Liggitt D, Yechan-Gunja S, Sarvothama M, Cooper K, Smythe KS, Dudakov JA, Pierce RH, Rader C, et al. Logic-Gated ROR1 Chimeric Antigen Receptor Expression Rescues T Cell-Mediated Toxicity to Normal Tissues and Enables Selective Tumor Targeting. *Cancer Cell*. 2019;35(3):489-503 e488.
7. Chu Y, Nayyar G, Tian M, Lee DA, Ozkaynak MF, Ayala-Cuesta J, Klose K, Foley K, Mendelowitz AS, Luo W, et al. Efficiently targeting neuroblastoma with the combination of anti-ROR1 CAR NK cells and N-803 in vitro and in vivo in NB xenografts. *Mol Ther Oncol*. 2024;32(2):200820.
8. Chu Y, Nayyar G, Jiang S, Rosenblum JM, Soon-Shiong P, Safrit JT, Lee DA, Cairo MS. Combinatorial immunotherapy of N-803 (IL-15 superagonist) and dinutuximab with ex vivo expanded natural killer cells significantly enhances in vitro cytotoxicity against GD2(+) pediatric solid tumors and in vivo survival of xenografted immunodeficient NSG mice. *J Immunother Cancer*. 2021;9(7).
