# Supplementary material for: Combinatorial immunotherapy with anti-ROR1 CAR NK cells and an IL-21 secreting oncolytic virus against neuroblastoma
Source: Mol Ther Oncol. 2024 Dec 21;33(1):200927. doi: 10.1016/j.omton.2024.200927 (PMC11783442; doi:10.1016/j.omton.2024.200927)
Supplement: Document S2. Article plus supplemental information [file mmc2.pdf]

Molecular Therapy
Oncology
Brief report

# Combinatorial immunotherapy with anti-ROR1 CAR NK cells and an IL-21 secreting oncolytic virus against neuroblastoma

Yaya Chu,[1,7] Meijuan Tian,[1,7] Uksha Saini,[2] Jessica Ayala-Cuesta,[1] Kayleigh Klose,[1] Alyssa S. Mendelowitz,[1] Keira Foley,[1] Mehmet F. Ozkaynak,[1] Wen Luo,[1] Timothy P. Cripe,[2] Dean A. Lee,[2] Kevin A. Cassady,[2,6] and Mitchell S. Cairo[1,3,4,5,6]

[1]Department of Pediatrics, New York Medical College, Valhalla, NY 10595, USA; [2]Center for Childhood Cancer and Blood Diseases, Nationwide Children's Hospital, The Ohio State University, Columbus, OH 43210, USA; [3]Department of Cell Biology & Anatomy, New York Medical College, Valhalla, NY 10595, USA; [4]Department of Pathology, Microbiology & Immunology, New York Medical College, Valhalla, NY, USA; [5]Department of Medicine, New York Medical College, Valhalla, NY 10595, USA

**Children with recurrent/metastatic neuroblastoma (NB) have a dismal survival (<25%). Novel therapies are desperately needed. Receptor tyrosine kinase-like orphan receptor 1 (ROR1) is highly expressed on NB. C021 is a selective oncolytic herpes simplex virus modified to overexpress human interleukin-21 (hIL-21), a cytokine that enhances natural killer (NK) cell cytotoxicity. In the current study, we successfully engineered *ex-vivo*-expanded NK cells to express a chimeric antigen receptor (CAR) against ROR1 using mRNA electroporation and investigated the efficacy of anti-ROR1-CAR-NK cells combined with C021 in targeting ROR1$^+$ NB. We found that C021-infected NB cells secreted hIL-21 *in vitro* and *in vivo*. Compared to the non-cytokine-secreting parental virus C134, C021 significantly enhanced the *in vitro* cytotoxicity ($p < 0.05$) of anti-ROR1-CAR-NK cells with increased interferon (IFN)-γ ($p < 0.05$), granzyme B ($p < 0.05$), and perforin ($p < 0.05$) secretion against NB cells. Furthermore, the combination of C021 and anti-ROR1-CAR-NK cells significantly extended the survival of human NB xenografted NSG mice compared to controls (mock NK, ROR1-CAR-NK, C134, C021, C134+ROR1-CAR-NK, and C021+mock NK). Our results suggest that cytokine-secreting oncolytic virus in combination with CAR-NK cells is a novel, effective immunotherapeutic approach for high-risk NB.**

## INTRODUCTION

Neuroblastoma (NB) stands as the most prevalent and fatal extracranial solid tumor affecting children, contributing to approximately 15% of childhood cancer fatalities.[1] Despite sustained efforts over several decades utilizing multi-chemotherapy, surgical resection, radiotherapy, autologous stem cell transplant, and targeted immunotherapy, survival for patients with high-risk NB remains dismal.[2] There is an urgent and unmet need for the development of novel treatments for these patients. Receptor tyrosine kinase-like orphan receptor 1 (ROR1) belongs to the receptor tyrosine kinase family[3] and has gained attention for its expression in several types of cancer, including NB, and its potential role as a therapeutic target.[4]

Natural killer (NK) cells provide the first line of defense against tumor cells.[5] Clinical trials (ClinicalTrials.gov: NCT02573896 and NCT04211675) are underway to assess the safety and efficacy of NK cell therapy alone or in combination with other modalities in treating patients with high-risk NB. However, NK therapy has current limitations, including small numbers of cells, poor cellular function, and/or decreased persistence *in vivo*.[6] Cytokines, however, have the potential to overcome these limitations by inducing proliferation and expansion, enhancing functional activity, and promoting persistence.

Interleukin-21 (IL-21) has the capability to promote the proliferation and function of NK cells.[7] In preclinical studies, IL-21 has demonstrated potent anti-tumor effects through mechanisms including the induction of interferon (IFN)-γ and the activation of NK and cytotoxic T cells.[8,9] Clinical trials have indicated promise for IL-21 as an immunotherapeutic agent in the treatment of patients with metastatic melanoma, showcasing both a favorable safety profile and notable anti-tumor activity.[10,11]

The oncolytic virus is a subtype of lytic virus designed to selectively infect and eliminate cancer but not normal cells.[12] Among the oncolytic viruses, oncolytic herpes simplex viruses (oHSVs) stand out, having advanced to phase 3 clinical trials.[12,13] oHSV possesses several therapeutic advantages, including its ability to infect various types of cancer cells and its ease of genetic modification.[14] The next-generation oHSV C134 has been genetically engineered to express a tumor-associated antigen (TAA), EphA2, to enhance TAA immune recognition and improve the anti-tumor activity of oHSV.[15]

Received 24 May 2024; accepted 18 December 2024;
https://doi.org/10.1016/j.omton.2024.200927.

[6]Senior author
[7]These authors contributed equally
**Correspondence:** Mitchell S. Cairo, Department of Pediatrics, New York Medical College, Valhalla, NY 10595, USA.
**E-mail:** mitchell_cairo@nymc.edu


© 2024 The Authors.
Published by Elsevier Inc. on behalf of The American Society of Gene and Cell Therapy.
This is an open access article under the CC BY license (http://creativecommons.org/licenses/by/4.0/).

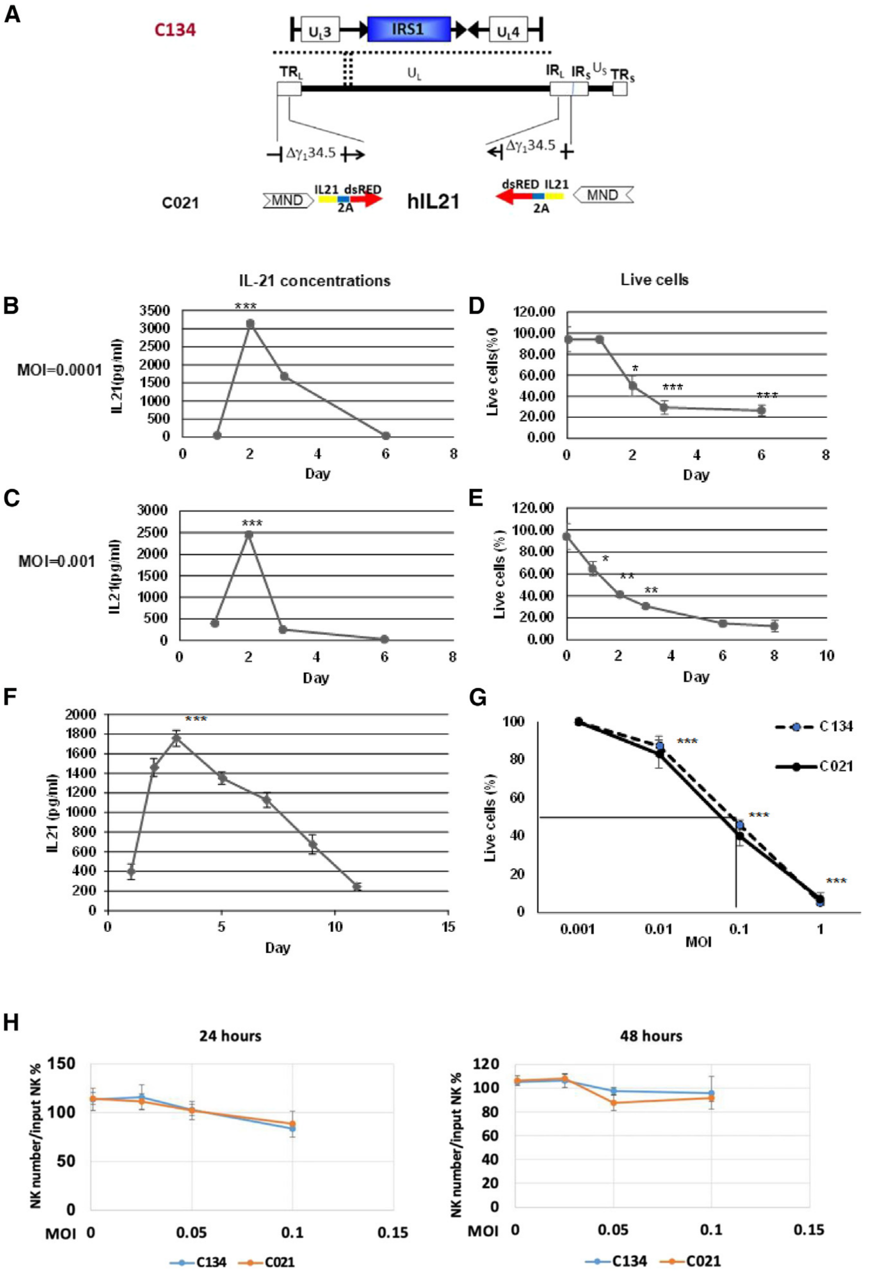

**Figure 1. The effects of C134 and C021 on NB cells and *ex-vivo*-expanded NK cells *in vitro***

(A) Schematic representation of C134 and C021 constructs. C021 contains the hIL-21 gene and dsRed (linked by a 2A element) within both copies of the $\gamma_1 34.5$ deletion locus of C134. (B) C021-infected CHLA-255 cells secreted significantly higher levels of hIL-21 on day 2 at MOI = 0.0001 ($p < 0.001$) compared to day 1, 3, or 6. $n = 3$. In this and the subsequent panels, markers represent the mean values, error bars indicate the standard deviation (SD) of triplicate samples in a representative experiment. The same trend was seen in three independent biological replicates. Results were compared using the two-tailed Student t-test with $p < 0.05$ considered as significant. (C) C021-infected CHLA-255 cells secreted significantly higher levels of hIL-21 on day 2 at MOI = 0.001 ($p < 0.001$) compared to day 1, 3, or 6. $n = 3$. (D) C021 significantly reduced the viability of CHLA-255 cells at day 2, 3, or 6 ($p < 0.001$) compared to day 1 at MOI = 0.0001. $n = 3$. (E) C021 significantly reduced the viability of CHLA-255 cells at day 2, 3, or 6 ($p < 0.001$) compared to day 1 at MOI = 0.001. $n = 3$. (F) At MOI = 0.025, C021-infected SKNFI cells secreted significantly higher levels of hIL-21 on day 3 ($p < 0.001$) compared to day 1, 2, 5, 7, 9, or 11. $n = 3$. (G) Both C134 and C021 infections significantly reduced the viability of SKNFI cells at 24 h at MOI = 0.01, 0.1, or 1 compared to MOI = 0.001 ($p < 0.001$). $n = 3$. (H) *Ex-vivo*-expanded NK cells were incubated with C134 or C021 at the indicated MOI. NK viability was measured at 24 and 48 h by CellTiter 96 AQueous one solution cell proliferation assay. $n = 3$. *$p < 0.05$ and ***$p < 0.001$.

In this study, we leveraged the C134-based virus and modified it to incorporate the expression of human IL-21 (hIL-21; C021). We hypothesized that intratumoral delivery of IL-21 by C021 would amplify the local anti-tumor efficacy of anti-ROR1-CAR-NK cells against NB.

## RESULTS

We modified C134 oncolytic virus to express hIL-21 (C021) (Figure 1A) and infected CHLA-255 NB cells with C021 utilizing a range of multiplicities of infection (MOIs) (Figures S1A–S1C). We found that infected CHLA-255 cells secreted significantly higher levels of hIL-21 at day 2 post-infection at MOI = 0.0001 (Figure 1B) ($p < 0.001$) or 0.001 (Figure 1C) ($p < 0.001$) compared to day 1, 3, or 6. In addition, C021 infection, at MOI = 0.0001 or 0.001, significantly reduced the viability of CHLA-255 cells ($p < 0.001$) (Figures 1D and 1E). Similarly, at MOI = 0.025, C021-infected SKNFI NB cells secreted higher levels of hIL-21 at day 3 post-infection ($p < 0.001$ compared to day 1, 2, 5, 7, 9, or 11) (Figure 1F), and C021 infection at MOI = 0.01, 0.1 or 1 significantly reduced the viability of SKNFI cells ($p < 0.001$ compared to MOI = 0.001) (Figure 1G). Notably, we observed no significant difference between C134 and C021 infections on the viability of SKNFI cells (Figure 1G). Neither C134 nor C021 reduced the viability of NK cells at the tested MOI (Figure 1H).

We next electroporated expanded NK cells[16] with anti-ROR1-CAR mRNA to generate anti-ROR1-CAR-NK cells (Figure S2) and investigated if C021 would enhance the *in vitro* cytotoxicity of anti-ROR1-CAR-NK cells against NB cells. Mock NK or anti-ROR1-CAR-NK cells were incubated with SKNFI cells at effector -to-target ratio

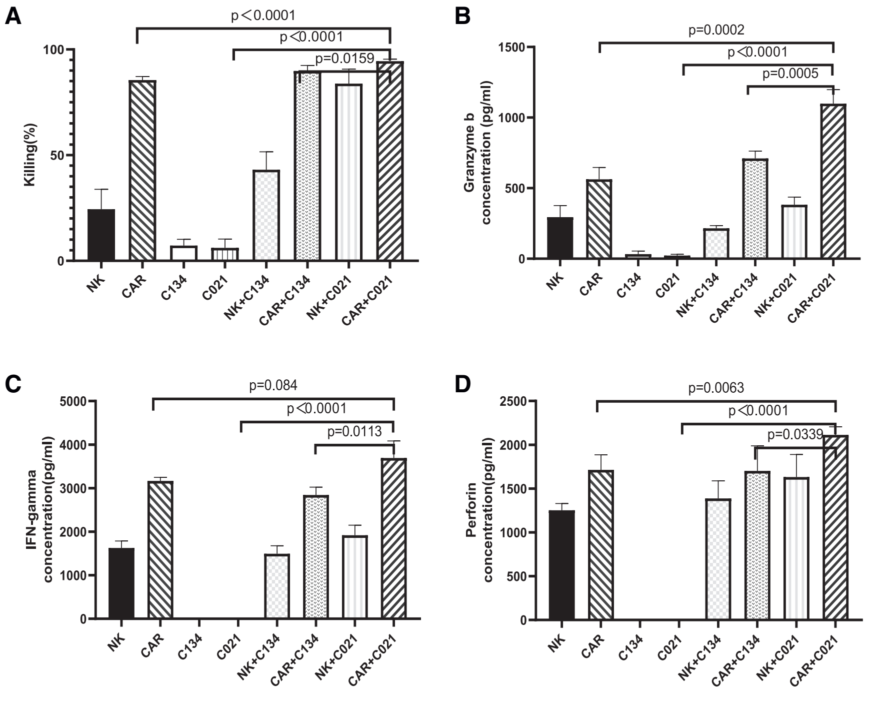


**Figure 2. C021 significantly enhanced *in vitro* cytotoxicity with the enhanced release of granzyme B, IFN-γ, and perforin of anti-ROR1-CAR-NK cells against NB cells**

(A) Mock NK or anti-ROR1-CAR-NK cells (CAR) were incubated with SKNFI cells at E:T = 3:1 with or without C134 or C021 (MOI = 0.025) for 24 h. The percentage of killing of SKNFI cells was measured by Britelite plus reporter gene assay. C021 significantly enhanced the *in vitro* cytotoxicity of anti-ROR1-CAR-NK cells against SKNFI cells compared to controls. In this and the subsequent panels, columns represent the mean values, error bars indicate the standard deviation (SD) of triplicate samples in a representative experiment. The same trend was seen in three independent biological replicates. Results were compared using the two-tailed Student t-test with $p < 0.05$ considered as significant. (B–D) After 24 h co-culture under the condition as described in (A), the supernatants were collected for ELISAs to determine the released granzyme B (B), IFN-γ (C), and perforin (D) levels. (E) Mock NK or anti-ROR1-CAR-NK cells (CAR) were incubated with CHLA-255 cells at E:T = 1:1 with or without C134 or C021 (MOI = 0.001) for 24 h. The percentage of killing of CHLA-255 cells was measured by Britelite plus reporter gene assay. C021 significantly enhanced the *in vitro* cytotoxicity of anti-ROR1-CAR-NK cells against CHLA-255 cells compared to controls ($p = 0.0286$ vs. CAR and CAR+C134, $p < 0.0001$ vs. C021). (F–H) After 24 h co-culture under the condition as described in (E), the supernatants were collected for ELISAs to determine the released granzyme B (F), IFN-γ (G), and perforin (H) levels. $n = 4$.

(E:T) = 3:1 with or without C134 or C021 (MOI = 0.025) for 24 h. C021 again induced IL-21 secretion (Figure S3) and significantly enhanced the *in vitro* cytotoxicity of anti-ROR1-CAR-NK cells against SKNFI cells compared to all controls (CAR-NK [$p < 0.0001$], C021 [$p < 0.0001$], CAR-NK+C134 [$p = 0.0159$]) (Figure 2A). Consistent with the enhanced *in vitro* cytotoxicity, we found that C021 significantly enhanced the secretion of granzyme B (Figure 2B) ($p = 0.0002$ vs. CAR, $p < 0.0001$ vs. C021, $p = 0.0005$ vs. CAR+C134), IFN-γ (Figure 2C) ($p = 0.084$ vs. CAR, $p < 0.0001$ vs. C021, $p = 0.0113$ vs. CAR+C134), and perforin (Figure 2D) ($p = 0.0063$ vs. CAR, $p < 0.0001$ vs. C021, $p = 0.0339$ vs. CAR+C134) compared to all controls. Similarly, C021 (MOI = 0.001) significantly enhanced the *in vitro* cytotoxicity of anti-ROR1-CAR-NK cells against CHLA-255 cells at an E:T ratio of 1:1 (Figure 2E) ($p < 0.0001$, $p = 0.0286$) with significantly enhanced secretion of granzyme B (Figure 2F) ($p < 0.0001$), IFN-γ (Figure 2G) ($p < 0.0001$, $p = 0.0001$, $p = 0.0385$), and perforin (Figure 2H) ($p < 0.0001$, $p = 0.0286$, $p = 0.0403$) compared to all controls.

Next, we investigated if C021-infected NB xenograft tumors would secrete hIL-21 *in vivo*. We injected CHLA-255 cells subcutaneously into NSG mice and injected C021 intratumorally once the tumor was established. We found that the tumors infected with $1 \times 10^4$ or $1 \times 10^5$ PFU/mouse C021 secreted significantly higher levels of hIL-21 ($p < 0.05$) compared to tumors infected with $1 \times 10^3$ PFU/mouse (Figure 3A), and the high IL-21 level in the tumors (Figure S4) lasted longer than in cell culture (Figure 1C).

To investigate if the combination of anti-ROR1-CAR-NK cells and C021 has advantages over anti-ROR1-CAR-NK cells alone or C021 alone in limiting NB tumor growth and improving mice survival, we xenografted luciferase-expressing CHLA-255 cells subcutaneously into NSG mice and treated the animals with PBS or $1 \times 10^4$ PFU C134 or C021 intratumorally (once) followed by intraperitoneal injection of PBS or $5 \times 10^6$ mock NK or ROR1-CAR-NK cells (twice: 2 and 9 days after viral injection) (Figures 3B and S5). The Kaplan-Meier survival curves showed that the mice treated with anti-ROR1-CAR-NK cells+C021 ($n = 5$) had significantly extended survival as compared to the control groups, which were treated with mock NK+C021 ($n = 5$, $p < 0.05$), anti-ROR1-CAR-NK cells alone ($n = 5$, $p < 0.05$), or C021 alone ($n = 5$, $p < 0.01$) (Figure 3C).

## DISCUSSION

Limited therapeutic options for high-risk NB highlight the pressing necessity for the development and implementation of innovative strategies to improve outcomes in this vulnerable patient population. In a parallel study, we demonstrated that ROR1 is highly expressed on NB and developed anti-ROR1-CAR-modified NK cells, and these anti-ROR1- CAR-NK cells showed significantly enhanced *in vitro* and *in vivo* anti-tumor effect against NB.[17] In the current study, our data demonstrated the anti-NB efficacy of the combination of an oncolytic virus engineered to secrete hIL-21 (C021) with anti-ROR1-CAR-NK cells.

Oncolytic viral therapy represents a promising avenue in the treatment of high-risk NB.[18] NB cell lines and human primary NB

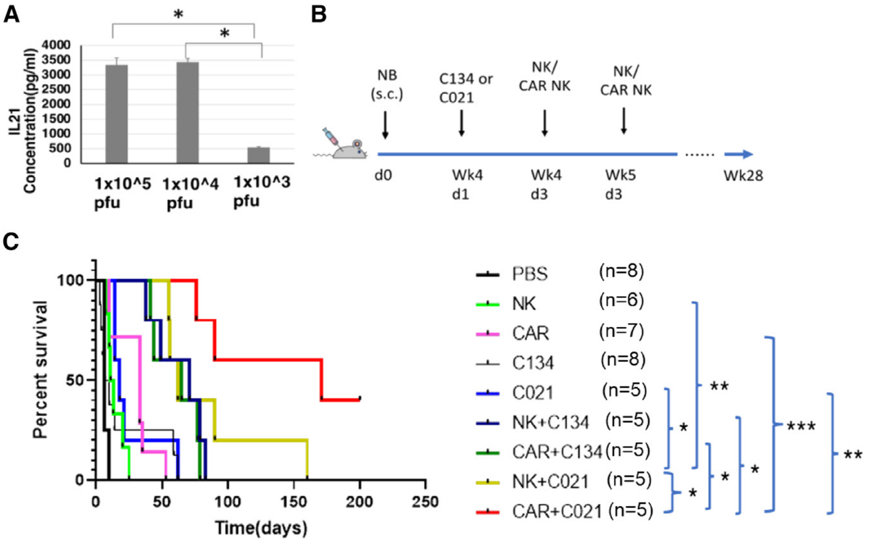


**Figure 3. The combination of anti-ROR1 CAR NK+C021 significantly extended the survival of NB xenografted NSG mice**

(A) hIL-21 expression in C021-injected NB xenograft tumors. 2 × $10^7$ of CHLA-255-Luc cells were subcutaneously injected into the right flank of NSG mice. After the tumor diameter reached 1 ± 0.3 cm, one dose of 1 × $10^3$, $10^4$, or $10^5$ PFU C021 was intratumorally injected into CHLA-255-Luc xenografted NSG mice. Two days later, tumors were collected and homogenized in 1 mL RPMI1640 medium. The tumor samples were centrifuged, and the supernatant was collected and used for IL-21 ELISAs. Columns represent the mean values, error bars indicate the standard deviation (SD) of all the samples. Results were compared using the two-tailed Student t-test with $p < 0.05$ considered as significant. (B) Experimental schema. 2 × $10^7$ of CHLA-255-Luc cells were subcutaneously injected into the right flank of NSG mice. After the tumor diameter reached 1 ± 0.3 cm, one dose of PBS or 1 × $10^4$ PFU C134 or C021 was intratumorally injected into CHLA-255-Luc xenografted mice. PBS, 5 × $10^6$ expanded peripheral blood natural killer (exPBNK) cells, or 5 × $10^6$ anti-ROR1-CAR-NK cells were intraperitoneally injected to each mouse 2 or 9 days after viral injection. (C) The Kaplan-Meier survival curves of mice receiving treatment are shown using animal sacrifice as the terminal event. Survival curves were analyzed using the log rank (Mantel-Cox) test. The mice treated with anti-ROR1-CAR-NK+C021 ($n = 5$) significantly extended the survival of CHLA255 xenografted mice as compared to the control groups, which were treated with mock NK+C021 ($n = 5$, $p < 0.05$), anti-ROR1-CAR-NK cells alone ($n = 5$, $p < 0.05$), or C021 alone ($n = 5$, $p < 0.01$). NK, mock NK; CAR, anti-ROR1-CAR-NK. *$p < 0.05$, **$p < 0.01$, and ***$p < 0.001$.

specimens expressed high levels of CD111, the primary entry protein of oHSV.[19] To reduce neurotoxicity and ensure replication in actively dividing cancer cells, but not normal (growth-arrested) cells, the $\gamma_1$34.5 gene, which encodes the protein 34.5 (ICP34.5), was deleted in C134 and C021.[13] This is consistent with our finding that both C134 and C021 efficiently lysed NB cells but not NK cells (Figure 1).

IL-21 shares the common γ chain as IL-2 and IL-15 and plays a crucial role in promoting NK proliferation and maturation from bone marrow.[20,21] The Lee laboratory developed a genetically engineered antigen-presenting cell (K562) expressing membrane-bound IL-21 and 4-1BBL (K562-mbIL21-41BBL) that expands NK cells out of peripheral blood mononuclear cells (PBMCs).[16] The donor-derived haploidentical NK cells expanded utilizing the K562-mbIL21-41BBL cells were safe, with significantly improved NK cell number and function, and improved survival in patients with myeloid malignancies.[22,23] Additionally, IL-21 helps sustain NK cell survival and memory-like responses, making it a key cytokine in modulating the overall effectiveness of NK cell-mediated immune responses.[24] IL-21 is critical to reverse the functions of exhausted NK cells.[25] To our knowledge, this is the first preclinical study investigating the therapeutic potential of the combination of anti-ROR1-CAR-NK cells with IL-21 intratumorally delivered by oncolytic viruses in treating high-risk NB.

Systemic delivery of IL-21 to NB may be limited by the inability to reach adequate concentrations of IL-21 across the entire tumor. Local delivery of IL-21 through oncolytic viruses offers several advantages, such as targeted delivery to tumor cells, reduced systemic toxicity, increased immune cell infiltration in tumor mass, enhanced therapeutic efficacy, a modified tumor microenvironment (TME), and immune-cell efficacy promotion.[12] Our data showed that C021 induced a high level of IL-21 secretion in the NB tumors, which sustained for around 6 days (Figure S4), demonstrating the successful local delivery of IL-21. Since C021 is modified to replicate specifically in tumor cells, this strategy facilitates efficient oncolysis of NB cells (Figure 1G) while minimizing probability of adverse off-target effects. Systemically delivered oncolytic viruses can be neutralized by the pre-existing or therapy-induced neutralizing antibodies; however, this risk can be minimized with local delivery. Although our *in vivo* study has a limitation in that it was investigated in a small set of animals, our finding that the combinatorial therapy of NK/anti-ROR1-CAR-NK cells with C021 significantly improved the survival of NB xenografted mice indicates that C021 shaped the TME and promoted NK/CAR-NK therapeutic efficacy. Further investigations with larger sample sizes will be necessary to prove this hypothesis.

In conclusion, our results demonstrate the significant anti-tumor efficacy of the combination of oHSV C021 with anti-ROR1-CAR-NK cells targeting NB cells *in vitro* and *in vivo*. Further research and clinical trials are essential to validate the safety and efficacy of this combination in treating high-risk NB.

## MATERIALS AND METHODS

### Anti-ROR1-CAR-NK generation

The anti-ROR1-CAR mRNA was synthesized *in vitro* using the mMESSAGE mMACHINE T7 Ultra kit as we previously described.[26]

Expanded NK cells were electroporated with anti-ROR1-CAR mRNA (1 μg mRNA per 1 × $10^6$ NK cells in EPB5 buffer) using the NK3 protocol of the MaxCyte GT electroporation System (Maxcyte, Rockville, MD, USA).

Additional methods are detailed in the supplemental information.

## DATA AND CODE AVAILABILITY

Data are available upon reasonable request.

## ACKNOWLEDGMENTS

The authors would like to thank Erin Morris, BSN, and Virginia Davenport, RN, for their excellent assistance with the preparation of this manuscript and Janet Ayello, MS, for her assistance with purchasing research reagents. The research for this study was primary supported by a grant from the NIH (1U54 CA232561-01A1; M.S.C., D.A.L., and T.P.C.) and additional support from the Pediatric Cancer Research Foundation (M.S.C.) and the Children Cancer Foundation (M.S.C.).

## AUTHOR CONTRIBUTIONS

Conceptualization, Y.C. and M.S.C.; visualization, Y.C. and M.T.; writing – original draft, Y.C., M.T., and M.S.C.; writing – review & editing, Y.C., M.T., U.S., J.A.-C., K.K., A.S.M., K.F., M.F.O., W.L., T.P.C., D.A.L., K.A.C., and M.S.C.; methodology, Y.C., M.T., U.S., and K.A.C.; formal analysis, Y.C., M.T., U.S., and K.A.C.; data curation, Y.C. and M.T.; validation, Y.C.; administrative, technical, and material support, T.P.C., D.A.L., K.A.C., and M.S.C.; funding, T.P.C., K.A.C., and M.S.C.

## DECLARATION OF INTERESTS

This work was presented in part at the Pediatric Transplantation & Cellular Therapy Consortium (2023), Fort Worth, TX; International Society for Cell & Gene Therapy (2022), San Francisco, CA; and Transplantation & Cellular Therapy Meetings of the American Society for Transplantation and Cellular Therapy (2022), Salt Lake City, Utah. M.S.C. has served as a consultant for Jazz Pharmaceuticals, Omeros Pharmaceuticals, Servier Pharmaceuticals, Abbvie, and Novartis Pharmaceuticals; on speakers bureau for Jazz Pharmaceuticals, Servier Pharmaceuticals, Amgen, Inc., Sanofi, and Sobi; and on the advisory board for Astra Zeneca and has received research funding from Celularity, Merck, Miltenyi Biotec, Servier, Omeros, Jazz, and Janssen. D.A.L. reports personal fees and other fees from Kiadis Pharma, CytoSen Therapeutics, Courier Therapeutics, and Caribou Biosciences outside the submitted work. In addition, D.A.L. has a patent broadly related to NK cell therapy of cancer with royalties paid to Kiadis Pharma. T.P.C. recently served as a one-time consultant to Blueprint, Incyte, and Oncopeptides and a DSMB chair for SpringWorks and is a cofounder of Vironexis Biotherapeutics, Inc.

## SUPPLEMENTAL INFORMATION

Supplemental information can be found online at https://doi.org/10.1016/j.omton.2024.200927.

## REFERENCES

1. Maris, J.M., Hogarty, M.D., Bagatell, R., and Cohn, S.L. (2007). Neuroblastoma. Lancet *369*, 2106–2120. https://doi.org/10.1016/S0140-6736(07)60983-0.

2. Smith, V., and Foster, J. (2018). High-Risk Neuroblastoma Treatment Review. Children *5*, 114. https://doi.org/10.3390/children5090114.

3. Kipps, T.J. (2022). ROR1: an orphan becomes apparent. Blood *140*, 1583–1591. https://doi.org/10.1182/blood.2021014760.

4. Shabani, M., Naseri, J., and Shokri, F. (2015). Receptor tyrosine kinase-like orphan receptor 1: a novel target for cancer immunotherapy. Expert Opin. Ther. Targets *19*, 941–955. https://doi.org/10.1517/14728222.2015.1025753.

5. Vivier, E., Raulet, D.H., Moretta, A., Caligiuri, M.A., Zitvogel, L., Lanier, L.L., Yokoyama, W.M., and Ugolini, S. (2011). Innate or adaptive immunity? The example of natural killer cells. Science *331*, 44–49. https://doi.org/10.1126/science.1198687.

6. Chu, Y., Gardenswartz, A., Termuhlen, A.M., and Cairo, M.S. (2019). Advances in cellular and humoral immunotherapy - implications for the treatment of poor risk childhood, adolescent, and young adult B-cell non-Hodgkin lymphoma. Br. J. Haematol. *185*, 1055–1070. https://doi.org/10.1111/bjh.15753.

7. Burns, L.J., Weisdorf, D.J., DeFor, T.E., Vesole, D.H., Repka, T.L., Blazar, B.R., Burger, S.R., Panoskaltsis-Mortari, A., Keever-Taylor, C.A., Zhang, M.J., and Miller, J.S. (2003). IL-2-based immunotherapy after autologous transplantation for lymphoma and breast cancer induces immune activation and cytokine release: a phase I/II trial. Bone Marrow Transplant. *32*, 177–186. https://doi.org/10.1038/sj.bmt.1704086.

8. Strengell, M., Matikainen, S., Sirén, J., Lehtonen, A., Foster, D., Julkunen, I., and Sareneva, T. (2003). IL-21 in synergy with IL-15 or IL-18 enhances IFN-gamma production in human NK and T cells. J. Immunol. *170*, 5464–5469. https://doi.org/10.4049/jimmunol.170.11.5464.

9. Eivary, S.H.A., Kheder, R.K., Najmaldin, S.K., Kheradmand, N., Esmaeili, S.A., and Hajavi, J. (2023). Implications of IL-21 in solid tumor therapy. Med. Oncol. *40*, 191. https://doi.org/10.1007/s12032-023-02051-4.

10. Thompson, J.A., Curti, B.D., Redman, B.G., Bhatia, S., Weber, J.S., Agarwala, S.S., Sievers, E.L., Hughes, S.D., DeVries, T.A., and Hausman, D.F. (2008). Phase I study of recombinant interleukin-21 in patients with metastatic melanoma and renal cell carcinoma. J. Clin. Oncol. *26*, 2034–2039. https://doi.org/10.1200/Jco.2007.14.5193.

11. Petrella, T.M., Tozer, R., Belanger, K., Savage, K.J., Wong, R., Smylie, M., Kamel-Reid, S., Tron, V., Chen, B.E., Hunder, N.N., et al. (2012). Interleukin-21 Has Activity in Patients With Metastatic Melanoma: A Phase II Study. J. Clin. Oncol. *30*, 3396–3401. https://doi.org/10.1200/Jco.2011.40.0655.

12. Lin, D., Shen, Y., and Liang, T. (2023). Oncolytic virotherapy: basic principles, recent advances and future directions. Signal Transduct. Targeted Ther. *8*, 156. https://doi.org/10.1038/s41392-023-01407-6.

13. Mamola, J.A., Chen, C.Y., Currier, M.A., Cassady, K., Lee, D.A., and Cripe, T.P. (2023). Opportunities and challenges of combining adoptive cellular therapy with oncolytic virotherapy. Mol. Ther. Oncolytics *29*, 118–124. https://doi.org/10.1016/j.omto.2023.04.008.

14. Zhu, X., Fan, C., Xiong, Z., Chen, M., Li, Z., Tao, T., and Liu, X. (2023). Development and application of oncolytic viruses as the nemesis of tumor cells. Front. Microbiol. *14*, 1188526. https://doi.org/10.3389/fmicb.2023.1188526.

15. Ghonime, M.G., Saini, U., Kelly, M.C., Roth, J.C., Wang, P.Y., Chen, C.Y., Miller, K., Hernandez-Aguirre, I., Kim, Y., Mo, X., et al. (2021). Eliciting an immune-mediated antitumor response through oncolytic herpes simplex virus-based shared antigen expression in tumors resistant to viroimmunotherapy. J. Immunother. Cancer *9*, e002939. https://doi.org/10.1136/jitc-2021-002939.

16. Denman, C.J., Senyukov, V.V., Somanchi, S.S., Phatarpekar, P.V., Kopp, L.M., Johnson, J.L., Singh, H., Hurton, L., Maiti, S.N., Huls, M.H., et al. (2012). Membrane-bound IL-21 promotes sustained ex vivo proliferation of human natural killer cells. PLoS One *7*, e30264. https://doi.org/10.1371/journal.pone.0030264.

17. Chu, Y., Nayyar, G., Wong, H.C., Seeger, R.C., Lee, J.H., Riddell, S.R., Safrit, J., Lee, D., and Cairo, M.S. (2020). Significant Targeting of Neuroblastoma By Anti-ROR1 Chimeric Antigen Receptor (CAR) Engineered NK Cells with or without IL-15 Superagonist (N-803) in Vitro and In Vivo Using Human Neuroblastoma Xenografted NSG Mice. Biol. Blood Marrow Transplant. *26*, S256. https://doi.org/10.1016/j.bbmt.2019.12.449.

18. Chen, X.T., Dai, S.Y., Zhan, Y., Yang, R., Chen, D.Q., Li, Y., Zhou, E.Q., and Dong, R. (2022). Progress of oncolytic virotherapy for neuroblastoma. Front. Pediatr. *10*, 1055729. https://doi.org/10.3389/fped.2022.1055729.

19. Gillory, L.A., Megison, M.L., Stewart, J.E., Mroczek-Musulman, E., Nabers, H.C., Waters, A.M., Kelly, V., Coleman, J.M., Markert, J.M., Gillespie, G.Y., et al. (2013). Preclinical evaluation of engineered oncolytic herpes simplex virus for the treatment of neuroblastoma. PLoS One *8*, e77753. https://doi.org/10.1371/journal.pone.0077753.

20. Asao, H., Okuyama, C., Kumaki, S., Ishii, N., Tsuchiya, S., Foster, D., and Sugamura, K. (2001). Cutting edge: the common gamma-chain is an indispensable subunit of the IL-21 receptor complex. J. Immunol. *167*, 1–5. https://doi.org/10.4049/jimmunol.167.1.1.

21. Parrish-Novak, J., Dillon, S.R., Nelson, A., Hammond, A., Sprecher, C., Gross, J.A., Johnston, J., Madden, K., Xu, W., West, J., et al. (2000). Interleukin 21 and its receptor

are involved in NK cell expansion and regulation of lymphocyte function. Nature *408*, 57–63. https://doi.org/10.1038/35040504.

22. Lee, D.A., Denman, C.J., Rondon, G., Woodworth, G., Chen, J., Fisher, T., Kaur, I., Fernandez-Vina, M., Cao, K., Ciurea, S., et al. (2016). Haploidentical Natural Killer Cells Infused before Allogeneic Stem Cell Transplantation for Myeloid Malignancies: A Phase I Trial. Biol. Blood Marrow Transplant. *22*, 1290–1298. https://doi.org/10.1016/j.bbmt.2016.04.009.

23. Ciurea, S.O., Kongtim, P., Soebbing, D., Trikha, P., Behbehani, G., Rondon, G., Olson, A., Bashir, Q., Gulbis, A.M., Indreshpal, K., et al. (2022). Decrease post-transplant relapse using donor-derived expanded NK-cells. Leukemia *36*, 155–164. https://doi.org/10.1038/s41375-021-01349-4.

24. Venkatasubramanian, S., Cheekatla, S., Paidipally, P., Tripathi, D., Welch, E., Tvinnereim, A.R., Nurieva, R., and Vankayalapati, R. (2017). IL-21-dependent expansion of memory-like NK cells enhances protective immune responses against Mycobacterium tuberculosis. Mucosal Immunol. *10*, 1031–1042. https://doi.org/10.1038/mi.2016.105.

25. Seo, H., Jeon, I., Kim, B.S., Park, M., Bae, E.A., Song, B., Koh, C.H., Shin, K.S., Kim, I.K., Choi, K., et al. (2017). IL-21-mediated reversal of NK cell exhaustion facilitates anti-tumour immunity in MHC class I-deficient tumours. Nat. Commun. *8*, 15776. https://doi.org/10.1038/ncomms15776.

26. Chu, Y., Hochberg, J., Yahr, A., Ayello, J., van de Ven, C., Barth, M., Czuczman, M., and Cairo, M.S. (2015). Targeting CD20+ Aggressive B-cell Non-Hodgkin Lymphoma by Anti-CD20 CAR mRNA-Modified Expanded Natural Killer Cells In Vitro and in NSG Mice. Cancer Immunol. Res. *3*, 333–344. https://doi.org/10.1158/2326-6066.CIR-14-0114.

OMTON, Volume *33*

## Supplemental information

# Combinatorial immunotherapy with anti-ROR1 CAR NK cells and an IL-21 secreting oncolytic virus against neuroblastoma

**Yaya Chu, Meijuan Tian, Uksha Saini, Jessica Ayala-Cuesta, Kayleigh Klose, Alyssa S. Mendelowitz, Keira Foley, Mehmet F. Ozkaynak, Wen Luo, Timothy P. Cripe, Dean A. Lee, Kevin A. Cassady, and Mitchell S. Cairo**

## Supplemental Methods

### Cell lines, viruses, and reagents

SKNFI cells were purchased from the American Type Culture Collection, Gaithersburg, MD, USA. The luciferase expressing CHLA-255 cells were generously provided by Robert Seeger, MD from Children's Hospital Los Angeles, CA, USA. Anti-ROR1 scFv was generously provided by Stanley Riddell, MD from Fred Hutchinson Cancer Center, Seattle, WA, USA. Leukocytes were obtained after informed consent from healthy donors at the New York Blood Center, New York, New York, USA. Peripheral blood mononuclear cells (PBMNC) were obtained by Ficoll gradient (Cytiva, Marlborough, MA, USA) separation as we previously described.[1] Recombinants C134 has been described previously.[2,3] Briefly, C134 is a $\Delta\gamma_1 34.5$ virus that contains the HCMV IRS1 gene under control of the CMV IE promoter in the $U_L3$/ $U_L4$ intergenic region and has been described previously.[2] C154 is an EGFP-expressing version of C134 with EGFP encoded in the $\gamma_1 34.5$ locus flanked by engineered Pac I restriction sites. C021 was created using the PacI homologous recombination method previously described (Figure 1A).[4] In brief the Pac I digested C154 viral DNA was co-transfected with linearized targeting plasmid pCK1238, encoding the Egr1 promoter driven hIL21 coding F2A and dsRED coding domain. C021 was then purified by loss of GFP and gain of dsRED expression by serial plaque selection. Viruses were confirmed genetically by DNA hybridization studies and IL21 expression was confirmed by ELISA.

### NK cell expansion

PBMNCs were stimulated with irradiated genetically modified K562-mbIL21- 41BBL cells as we previously described.[5] Expanded NK cells were isolated by negative selection using Miltenyi

NK cell isolation kit (Miltenyi Biotec, Cambridge, MA, USA) as we have previously described.[1] Expanded purified NK cells were cultured in Gibco RPMI 1640 medium (Thermofisher) supplemented with 10% heat-inactivated FBS (Thermofisher), 100 U/mL penicillin, 100 μg/mL streptomycin (Thermofisher), 4 mmol/L glutamine (Thermofisher), and 50 IU/ml IL-2.

**Anti-ROR1-CAR-NK generation**

The ROR1 CAR was generously provided by Stanley Riddell MD (Fred Hutchinson Cancer Research Center) and was previously reported.[6,7] The CAR possessed a murine CD8α signal peptide (UniProt: P01731, aa1–27), R11 scFv, modified human IgG4 long spacer with 4/2NQ mutations (Hudecek et al., 2015), murine CD28 transmembrane (UniProt: P31041, aa151–177), murine 4–1BB (UniProt: P20334, aa211–256), murine CD3ζ (UniProt: P24161, aa52–164). The anti-ROR1-CAR mRNA was synthesized *in-vitro* using the mMESSAGE mMACHINE T7 Ultra kit as we previously described.[1] Expanded NK cells were electroporated with anti-ROR1-CAR mRNA using the MaxCyte GT® electroporation System (Maxcyte Inc., Rockville, MD, USA). Anti-ROR1-CAR mRNA electroporation efficacy was evaluated by flow cytometry analysis using an FITC-conjugated goat anti-mouse IgG, $F(ab')_2$ fragment-specific antibody. Expanded NK cells electroporated with H2O (mock NK) were used as a control.

**Luciferase based *in-vitro* cytotoxicity**

Expanded NK or anti-ROR1-CAR-NK cells were incubated with SKNF1-Luc cells at the effector:target (E:T) ratio=3:1 with or without C134 or C021 (MOI = 0.025) at 37$^{o}$C for overnight. NK or anti-ROR1-CAR-NK cells were incubated with CHLA -255-Luc cells E:T ratio=1:1 with or without C134 or C021 (MOI = 0.001) in RPMI media at 37$^{o}$C for overnight.

Cytotoxicity was determined by Britelite plus reporter gene assay (PerkinElmer, 6066761) without cell lysis. After adding luciferin, the luminescence emission of viable cells was measured by a plate reader (Molecular devices, Filter max F5 microplate reader) within 15 minutes. Cytotoxicity was calculated as follows: % cytotoxicity= (Luminescence release (untreated tumor cells)- Luminescence release (treated tumor cells)) / Luminescence release (untreated tumor cells)) ×100.

**MTS assays**

PBMNCs were stimulated with irradiated genetically modified K562-mbIL21 - 41BBL cells for 2-3 weeks. Purified expanded NK cells were cultured in medium with C134 or C021 at different MOI for 24 hours or 48 hours. CellTiter 96 AQueous one solution cell proliferation assay (Promega, Madison, WI, USA) was used to determine the number of proliferating viable cells following the manufacturer instructions as we previously described.[8] Spectrophotometrical absorbance was measured using a multifilter plate reader (Molecular Device, San Jose, CA, USA) at OD490.

**Enzyme-linked immunosorbent assay (ELISA)**

Human granzyme B (Mabtech Cat#3485-1H-20), perforin (Mabtech Cat#3465-1H-20), or IFN-γ (Mabtech Cat#3420-1H-20) were measured by ELISA assays. Greiner Bio-One 96 well high binding standard ELISA microplates (Cat# 655061) were coated with 100 µl of human granzyme B, perforin, or IFN-γ (2 µg/ml ,4 µg/ml, 2µg/ml in phosphate-buffered saline (PBS), respectively) by incubating plates overnight at 4 °C. Wells were then washed twice with 200 µl wash buffer (0.05% Tween-20 in PBS) and blocked with 250 µl blocking buffer (0.1% BSA in

PBS) for 1 hour at room temperature. Samples or standards were added to the wells (100 µl per well) in technical duplicates or triplicates, and plates were incubated for 2 hours at room temperature and washed three times. Biotin conjugated antibodies (1µg/ml, 100 µl) were added and incubated for 1 hour at room temperature. Following 3 times of wash, 100 µl of horseradish peroxidase- diluted 1:1,000 was added to the wells and plates were incubated for 1 hour at room temperature. The wells were washed three times and incubated with 100 µl of TMB substrate solution (Invitrogen, Waltham, MS, USA) for 2-5 mins. The reaction was stopped by adding 100 µl of 2 N sulfuric acid, and plates were read at 450 nm using the FilterMax F5 (Molecular devices) plate reader.

**Xenograft models**

Six- to eight-week-old NSG mice were purchased from the Jackson Laboratory (Bar Harbor, ME, USA). Mice were bred, treated, and maintained under pathogen-free conditions in-house under New York Medical College Institutional Animal Care and Use Committee-approved protocols. CHLA-255-Luc cells ($2x10^7$) were subcutaneously injected to the right flanks of NSG mice. After the tumor diameter reached 1±0.3 cm, the mice were separated into 9 groups: 1) Mock NK, 2) anti-ROR1-CAR-NK, 3) C134, 4) C021, 5) Mock NK+ C134, 6) Mock NK+C021, 7) anti-ROR1-CAR-NK+C134, 8) anti-ROR1-CAR-NK+C021, and 9) PBS. $1x10^4$ pfu C134 or C021 or PBS was intratumorally injected on day 1 of the treatment (7 days after tumor cell injection). Two days and nine days later, $5x10^6$ NK or anti-ROR1-CAR-NK cells or PBS were intraperitoneally injected. Tumor engraftment and progression were evaluated using the Xenogen IVIS-200 system (PerkinElmer, Shelton, CT, USA) as we have previously described.[1] Tumor

size was measured by a caliper. Mice were followed until death or sacrificed if the diameter of tumor reached 2 cm.

**Statistical analyses**

Statistical analyses were performed using the Prism program 10.0 (GraphPad Software, Inc.). Average values are reported as the mean ± SD. Results were compared using the two-tailed Student t-test with $p < 0.05$ considered as significant. Probability of survival in animal studies was determined by the Kaplan-Meier method using the Prism program 10.0 (GraphPad Software, Inc., La Jolla, CA, USA). Survival rates were compared using the log-rank Mantel–Cox test.

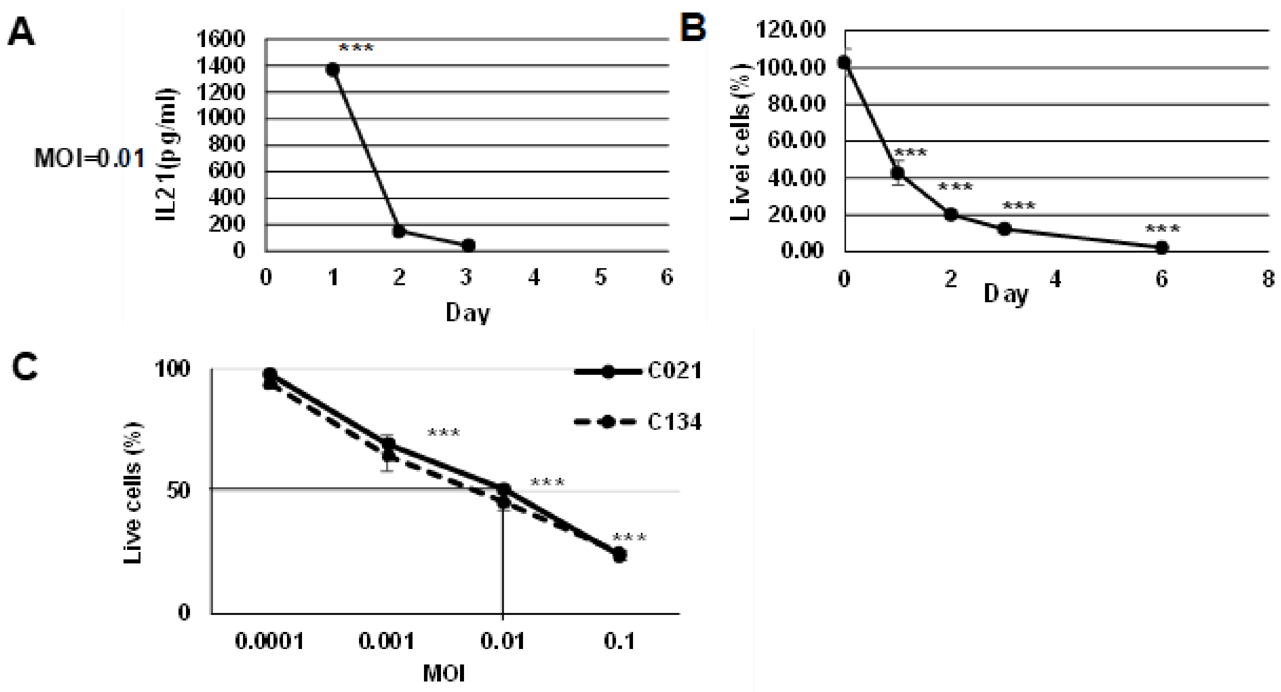


**Figure S1. The effects of C134 and C021 on NB cells and ex-vivo expanded NK cells in-vitro.** (A) C021 infected CHLA-255 cells secreted significantly higher level of hIL-21 at day 1 at MOI = 0.01 ($p < 0.001$) compared to day 2 or day 3. n = 3 (B) C021 significantly reduced the viability of CHLA-255 cells at day 2, 3 or 6 ($p < 0.001$) compared to day 1 at MOI = 0.01. n = 3. (C) Both C134 and C021 infection significantly reduced the viability of CHLA-255 cells at 24 hours at MOI = 0.001, 0.01 or 0.1 compared to MOI = 0.0001 ($p < 0.001$). n = 3

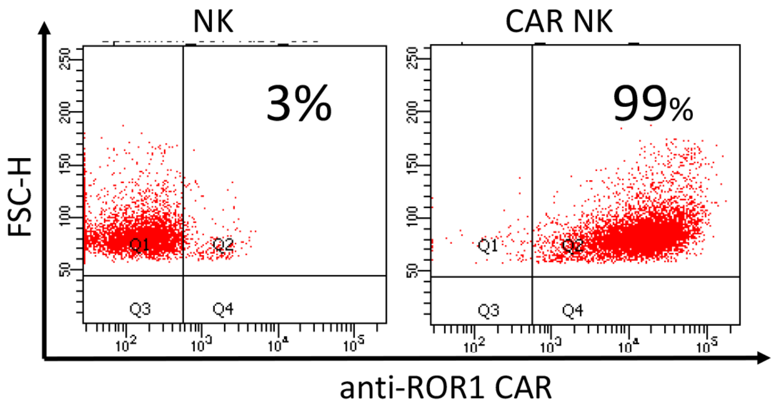


**Figure S2. Representative flow cytometry dot plots show the anti-ROR1 CAR expression.** The anti-ROR1 CAR mRNA or the anti-ROR1 CAR-CD19 mRNA was synthesized *in vitro* using the mMESSAGE mMACHINE T7 Ultra kit. Expanded NK cells were electroporated with anti-ROR1 CAR mRNA or the anti-ROR1 CAR-CD19 mRNA using the MaxCyte GT® electroporation System (Maxcyte Inc.). Anti-ROR1 CAR mRNA electroporation efficacy was evaluated by flow cytometry analysis at 24 hours post electroporation using an FITC-conjugated goat anti-mouse IgG, $F(ab')_2$ fragment-specific antibody. Expanded NK cells electroporated with H2O (mock NK) were used as control.

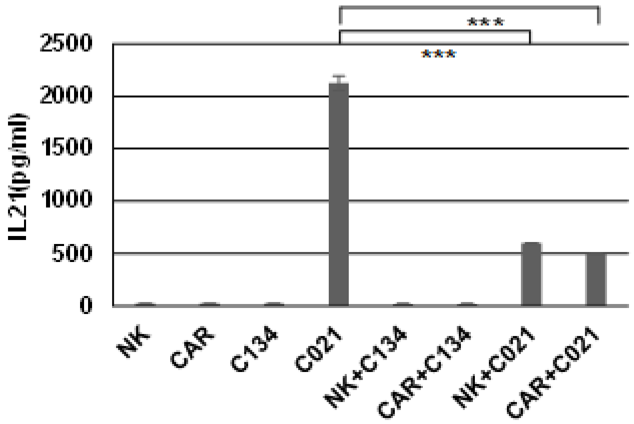


**Figure S3. C021 release was influenced by cytotoxicity of NK or anti-ROR1-CAR-NK cells (CAR) cells.** Mock NK or anti-ROR1-CAR-NK cells (CAR) were incubated with CHLA-255 cells at E:T=1:1 with or without the pretreatment of C134 or C021 (MOI = 0.001) for 24 hours. The supernatants were collected for ELISA assays to determine the hIL-21 release in each group.

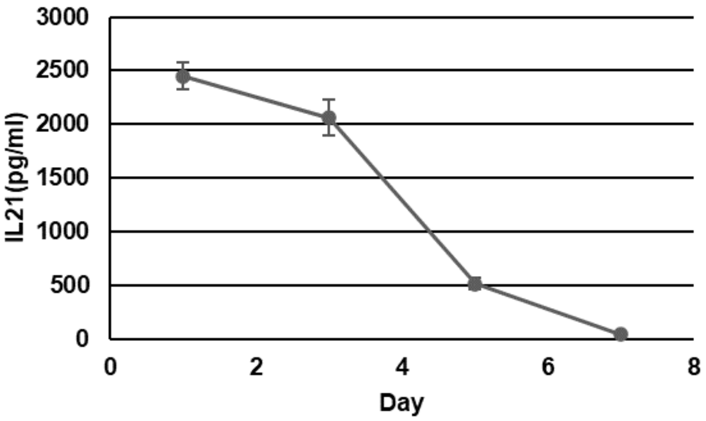


**Figure S4. C021 infected NB cells in NB xenografts secreted hIL21.** CHLA-255-Luc cells ($2x10^7$) were subcutaneously injected to the right flank in NSG mice. After the tumor diameter reached 1±0.3 cm, one dose of $1x10^4$ pfu C021 was intratumorally injected to the CHLA-255-Luc xenografted NSG mice. Tumors were collected and homogenized in 1ml RPMI1640 medium on day 1,3,5 and 7. The tumor samples were centrifuged, and the supernatant was collected and used for IL-21 ELISA assays. N=3.

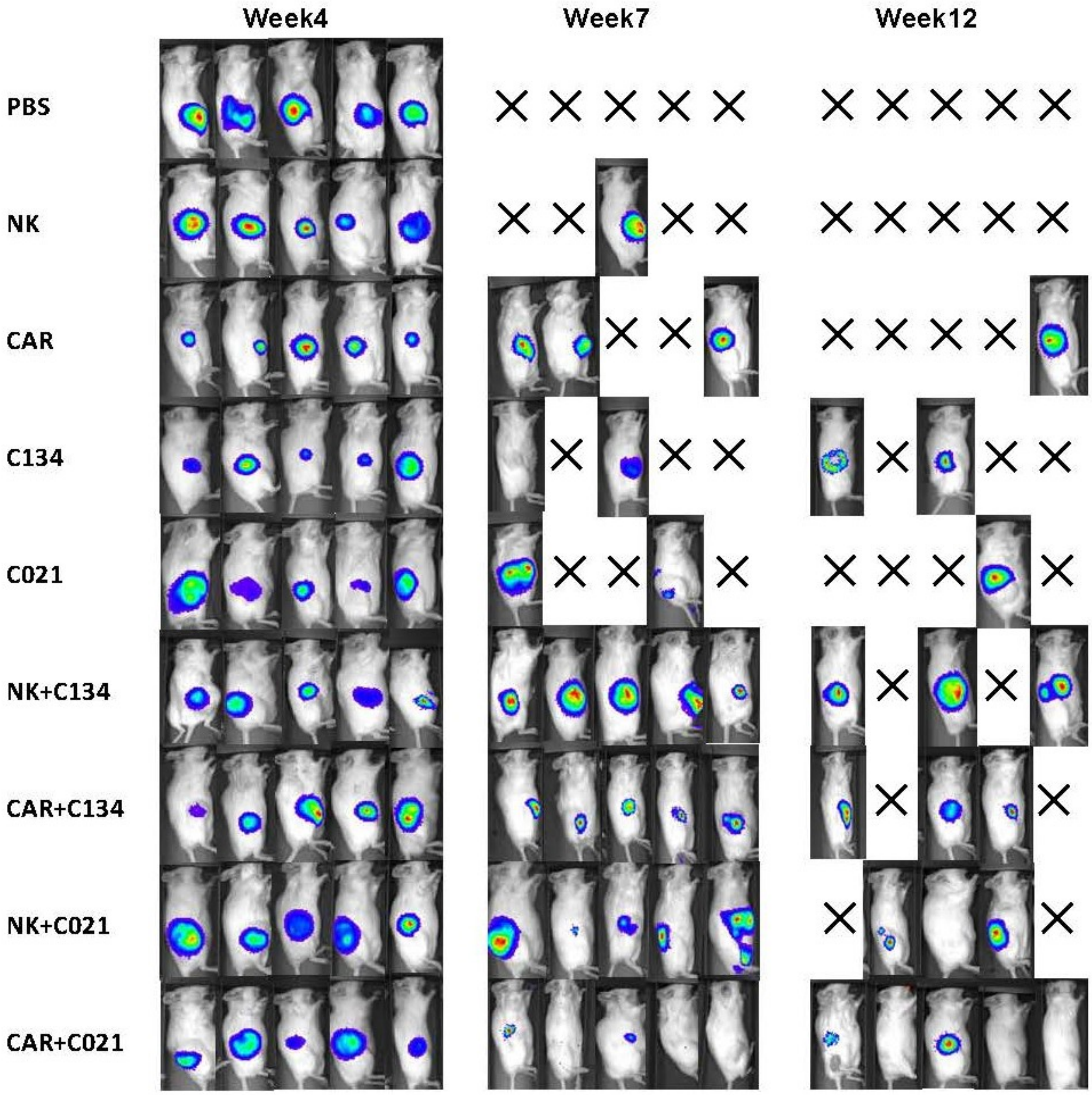


**Figure S5. The combination of anti-ROR1 CAR NK + C021 significantly extended the survival of NB xenografted NSG mice.** CHLA-255-Luc cells ($2x10^7$) were subcutaneously injected to the right flank in NSG mice. After the tumor diameter reached 1±0.3 cm, one dose of PBS, 1x104 pfu HSV virus or $5x10^6$ exPBNK /CAR cells were injected as mentioned in Fig.4. Representative bioluminescence images of mice of each group are shown at week4 (one day before treatment), week 7(2week post treatment) and week 12.

REFERENCES


1. Chu Y, Hochberg J, Yahr A, Ayello J, van de Ven C, Barth M, Czuczman M, Cairo MS. Targeting CD20+ Aggressive B-cell Non-Hodgkin Lymphoma by Anti-CD20 CAR mRNA-Modified Expanded Natural Killer Cells In Vitro and in NSG Mice. *Cancer Immunol Res*. 2015;3(4):333-344.
2. Cassady KA. Human cytomegalovirus TRS1 and IRS1 gene products block the double-stranded-RNA-activated host protein shutoff response induced by herpes simplex virus type 1 infection. *J Virol*. 2005;79(14):8707-8715.
3. Ghonime MG, Jackson J, Shah A, Roth J, Li M, Saunders U, Coleman J, Gillespie GY, Markert JM, Cassady KA. Chimeric HCMV/HSV-1 and Deltagamma(1)34.5 oncolytic herpes simplex virus elicit immune mediated antigliomal effect and antitumor memory. *Transl Oncol*. 2018;11(1):86-93.
4. Parker JN, Zheng X, Luckett W, Markert JM, Cassady KA. Strategies for the rapid construction of conditionally-replicating HSV-1 vectors expressing foreign genes as anticancer therapeutic agents. *Mol Pharm*. 2011;8(1):44-49.
5. Denman CJ, Senyukov VV, Somanchi SS, Phatarpekar PV, Kopp LM, Johnson JL, Singh H, Hurton L, Maiti SN, Huls MH, et al. Membrane-bound IL-21 promotes sustained ex vivo proliferation of human natural killer cells. *PLoS One*. 2012;7(1):e30264.
6. Srivastava S, Salter AI, Liggitt D, Yechan-Gunja S, Sarvothama M, Cooper K, Smythe KS, Dudakov JA, Pierce RH, Rader C, et al. Logic-Gated ROR1 Chimeric Antigen Receptor Expression Rescues T Cell-Mediated Toxicity to Normal Tissues and Enables Selective Tumor Targeting. *Cancer Cell*. 2019;35(3):489-503 e488.
7. Chu Y, Nayyar G, Tian M, Lee DA, Ozkaynak MF, Ayala-Cuesta J, Klose K, Foley K, Mendelowitz AS, Luo W, et al. Efficiently targeting neuroblastoma with the combination of anti-ROR1 CAR NK cells and N-803 in vitro and in vivo in NB xenografts. *Mol Ther Oncol*. 2024;32(2):200820.
8. Chu Y, Nayyar G, Jiang S, Rosenblum JM, Soon-Shiong P, Safrit JT, Lee DA, Cairo MS. Combinatorial immunotherapy of N-803 (IL-15 superagonist) and dinutuximab with ex vivo expanded natural killer cells significantly enhances in vitro cytotoxicity against GD2(+) pediatric solid tumors and in vivo survival of xenografted immunodeficient NSG mice. *J Immunother Cancer*. 2021;9(7).
